# Epistatic interactions inform rational design of synthetic microbial communities for bioremediation

**DOI:** 10.1038/s41564-026-02386-4

**Published:** 2026-06-29

**Authors:** Mahmoud Yousef, Kiseok Keith Lee, Junivere Tang, Paige Mullen, Vasileios Charisopoulos, Rebecca Willett, Seppe Kuehn

**Affiliations:** 1 Medical Scientist Training Program, The University of Chicago Pritzker School of Medicine, Chicago, IL USA; 2 Department of Ecology and Evolution, The University of Chicago, Chicago, IL USA; 3 Center for the Physics of Evolving Systems, The University of Chicago, Chicago, IL USA; 4 Center for Living Systems, The University of Chicago, Chicago, IL USA; 5 Data Science Institute, The University of Chicago, Chicago, IL USA; 6 National Institute for Theory and Mathematics in Biology, Northwestern University and The University of Chicago, Chicago, IL USA

**Keywords:** Biophysics, Applied microbiology, Community ecology

## Abstract

The metabolism of microbial communities is essential for host and environmental health. The rational design of microbiomes with targeted functional properties is an important objective but remains challenging due to complex interactions and environmental heterogeneity. Community–function landscapes address this challenge by statistically inferring impacts of species presence or absence on function. Similar to fitness landscapes, community–function landscapes estimate both additive effects and interactions (epistasis) between species that influence function. We apply landscapes to design synthetic consortia to degrade the toxic contaminant bisphenol-A (BPA). Using synthetic communities of ten BPA-degrading bacteria, we map community–function landscapes across increasing BPA concentrations, where higher BPA means greater toxicity. Epistasis increases with toxicity, indicating that collective effects become more important for degradation. Designed communities are able to remediate BPA in contaminated soils. Our results demonstrate that toxicity can drive epistatic interactions in community–function landscapes and that these landscapes can guide microbial consortia design for bioremediation.

## Abstract (web summary)

By taking into account the impacts of species presence, absence and potential interactions, synthetic soil microbial consortia are designed to degrade the toxic environmental contaminant bisphenol-A.

## Main

Microbial communities drive processes essential for human and environmental health. Efforts to recruit microbial communities with desirable properties^1–3^ have seen mixed success. These challenges have driven interest in synthetic ecology: engineering communities with desirable functional properties. However, a community’s function is affected by a myriad of factors, including gene expression^4^, ecological interactions^5–7^ and environmental variables^8^. As a result, dissecting the relationship between the species present, their abundances and community function is a challenge.

One way to overcome this challenge is to take advantage of a conceptual analogy with fitness landscapes. In genetics, fitness landscapes map a genotype to an organism’s fitness^9^. Fitness is often impacted by interactions between individual components (for example, genes), not only their additive impacts. Fitness landscapes characterize interactions by measuring epistasis: the effect of multiple components that deviates from individual additive effects^9,10^. In the context of microbial communities, epistasis measures the impact of strains on community function beyond their individual contributions^11,12^. By exploiting community–function landscapes, maps between community composition and function^7,11,13^, it is possible to accurately predict how community function depends on composition^11^. However, with few exceptions^14^, most work inferring community–function landscapes targets simple synthetic communities^15^ and does not solve applied problems in biotechnology.

One biotechnological process that could benefit from rational community design is bioremediation. In this context, dangerous chemicals that contaminate water and soil can be remediated by the metabolic activity of microbes^16–20^. Bisphenol-A (BPA) is a widely used industrial chemical and pollutant^21,22^ whose removal offers substantial health benefits^23^. Although many bacteria catabolise BPA aerobically^24–28^, many strains fail to achieve complete degradation^27^ due to the toxicity of BPA degradation by-products^29–33^. The degradation pathway remains poorly characterized^24–26,31,34,35^, limiting rational routes to selecting strains for building communities resistant to BPA toxicity, with only modest successes reported^36^.

Here we address these challenges by exploiting a community–function landscape approach to design BPA-degrading communities across a range of BPA concentrations. Inferred landscapes show that collective effects, which are evident as epistasis, increase with the concentration of BPA. We find that the structure of the landscape changes smoothly as the BPA concentration rises. We use landscapes to design communities with desired rates of BPA degradation in vitro, and then show that designed communities remediate BPA in contaminated soils.

## Results

### A library of isolates to enable community design for remediation

**Extended Data Fig. 1:**
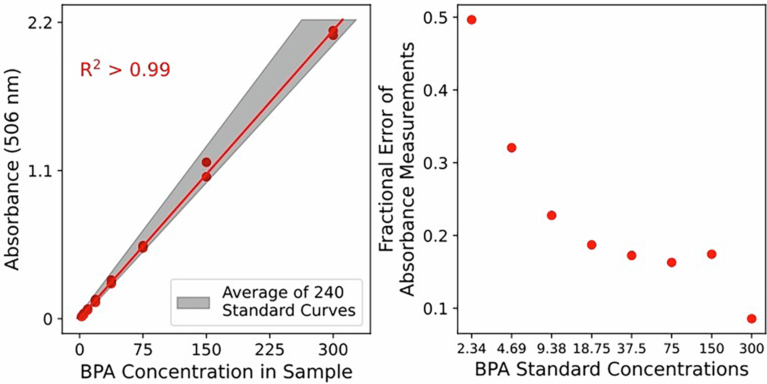
Standard Curve of BPA Concentration Assay. Left: A sample standard curve of BPA concentration, along with the line of best fit (red). R^2^ = 0.9998 as calculated by the scikit-learnfunction r2_score_. Gray shaded region indicates the error of the slope of the average standard curve (see Methods). Right: The fractional error of absorbance measurement as a function of the prepared BPA standard concentration. Fractional error is large at the lowest concentrations and small at the highest concentrations. Source data

**Extended Data Fig. 2:**
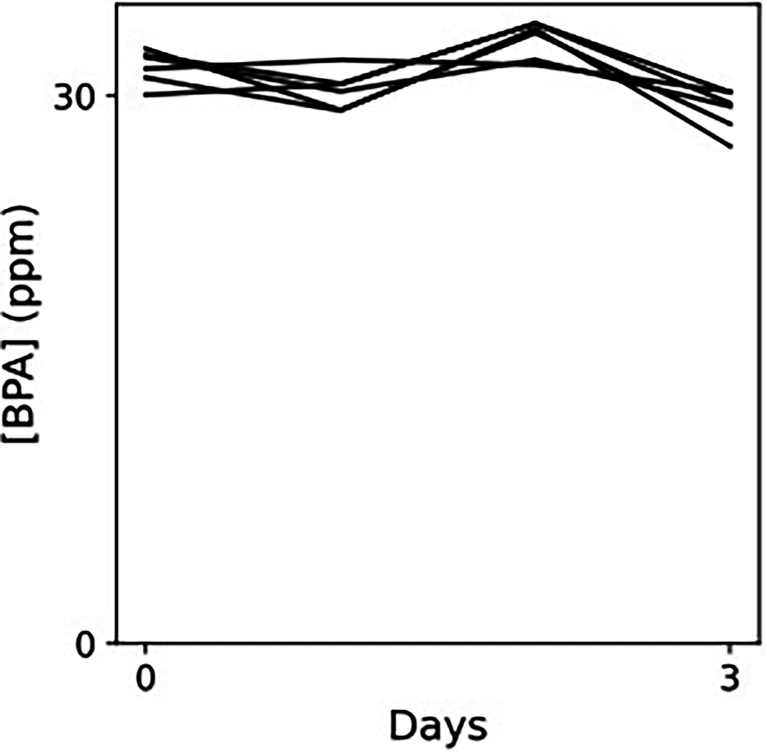
Random communities of soil isolates do not degrade BPA. 5 random communities were assembled from six Proteobacterial strains isolated from various forests and plains within Illinois. These strains have been previously characterized for denitrification (ref. ^45^). Communities were inoculated in 30 ppm BPA media, and the BPA concentration was measured every 24 hours for each community as described in the Methods section. Source data

We assembled communities from a library of soil isolates available in our laboratory^37^ (Proteobacteria) and tested their ability to degrade BPA in minimal media, assayed colorimetrically in time (Methods and Extended Data Fig. 1). None degraded BPA (Extended Data Fig. 2), indicating that BPA degradation is not a common trait among readily cultured soil isolates.

**Fig. 1:**
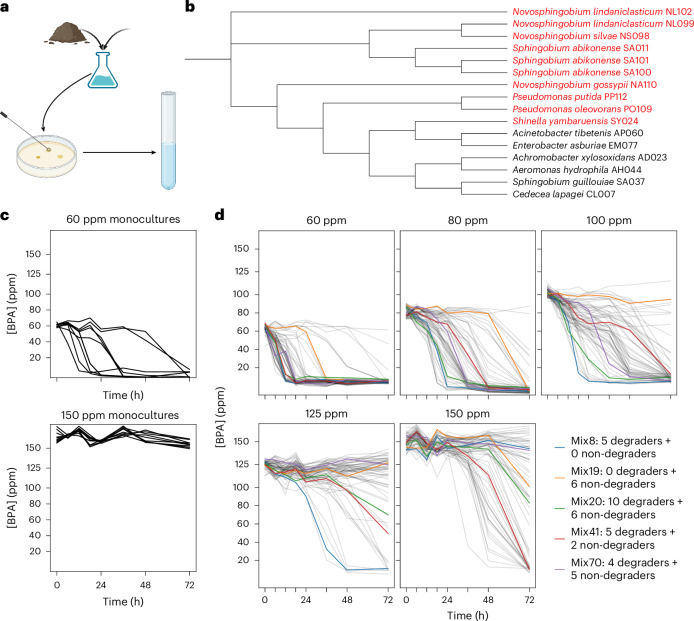
Isolates enable construction of synthetic bacterial communities that degrade BPA across varying initial concentrations. **a**, Protocol for enrichment of BPA degraders from soil. After enrichment, isolates are streaked to purity and screened for BPA degradation. Created with BioRender.com. **b**, Phylogenetic tree of the 16 strains isolated. Red strains denote strains that degrade BPA on their own from an initial concentration of 30 ppm. Note that 4 of 16 strains were not fully purified despite streaking as noted in Extended Data Table 1 (see text for discussion). **c**, Monocultures of the 10 BPA-degrading strains in an initial BPA concentration of 60 ppm (top) and 150 ppm (bottom). None of the monocultures degraded BPA at 150 ppm, indicating that degradation from high concentrations probably requires a consortium. **d**, BPA degradation curves of 70 communities in five initial concentrations of BPA. Five communities are highlighted: Mix8 (blue), Mix19 (yellow), Mix20 (green), Mix41 (red) and Mix70 (purple). All measurements are corrected for evaporation (Methods). Panel **a** created with BioRender.com. Source data

**Extended Data Fig. 3:**
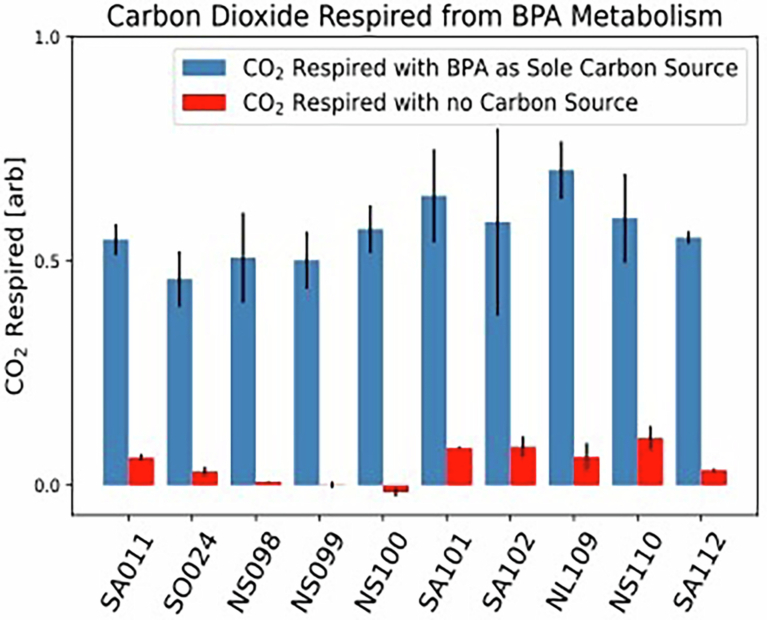
CO_2_ production by ten degraders during BPA degradation. Net CO2 production by the 10 BPA-degrading strains when individually inoculated in 60 ppm BPA (blue) and when inoculated in minimal media with no carbon source (red) for three days. Error bars represent the standard deviation of technical duplicates. CO2 measurements were performed with the microresp platform as described in the Methods section. Source data

We sought to construct a strain bank of bacterial isolates that could be used to assemble synthetic communities targeted for BPA degradation. We identified and sampled five sites with potential BPA contamination (see Methods and Supplementary Table 1, rows 1–5) and enriched each soil sample for BPA degradation (see Methods). From these samples, we isolated and identified 16 isolates, 10 of which degrade BPA from 30 ppm in monoculture (degraders) and 6 that did not (non-degraders) but were retained due to co-isolation with degraders (Fig. 1b and Extended Data Table 1). We confirmed that BPA degradation occurs via aerobic respiration by measuring CO_2_ production for each degrading isolate (Extended Data Fig. 3). Isolate purity was tested through long-read whole-genome sequencing (Methods); contamination was detected in 4 of the 16 isolates (Extended Data Table 1), indicating that these were not strictly pure cultures. Despite this, the community–function landscape approach enabled us to treat each isolate, pure or not, as a single unit (Methods).

By growing each strain in minimal medium with BPA as the sole carbon source, we measured degradation dynamics of each degrader at a low BPA concentration (60 ppm) and a highly toxic concentration (150 ppm). We found that while individual strains could degrade BPA at 60 ppm, no single isolate could degrade at 150 ppm (Fig. 1c). The result suggests that BPA toxicity at higher concentrations requires a consortium for degradation.

### Structure of community-level degradation with increasing toxicity

With our library of isolates at hand, we next set out to assemble synthetic communities and test their BPA degradation capabilities. Previous studies^14^ suggest that assembling diverse communities and quantifying their function can enable statistical design. This approach works especially well as predicting community function requires primarily estimating the average effect of each strain on function^11^.

Therefore, we assembled 70 communities from our 16 isolates of richness levels from 2 to 16 and inoculated them into BPA medium (see Methods). To assemble communities, each bacterial isolate was grown individually, and then the appropriate strains were combined with a fixed initial optical density (OD) of 0.02 for each strain, irrespective of community richness. We did not fix total initial biomass—thus, avoiding dilution of each strain at higher richness—because our statistical framework estimates each strain’s average contribution to community function. Fixing total biomass would force the per-strain inoculum to decrease as richness increased, confounding those estimates by making each strain’s apparent contribution decline with community richness.

**Extended Data Fig. 4:**
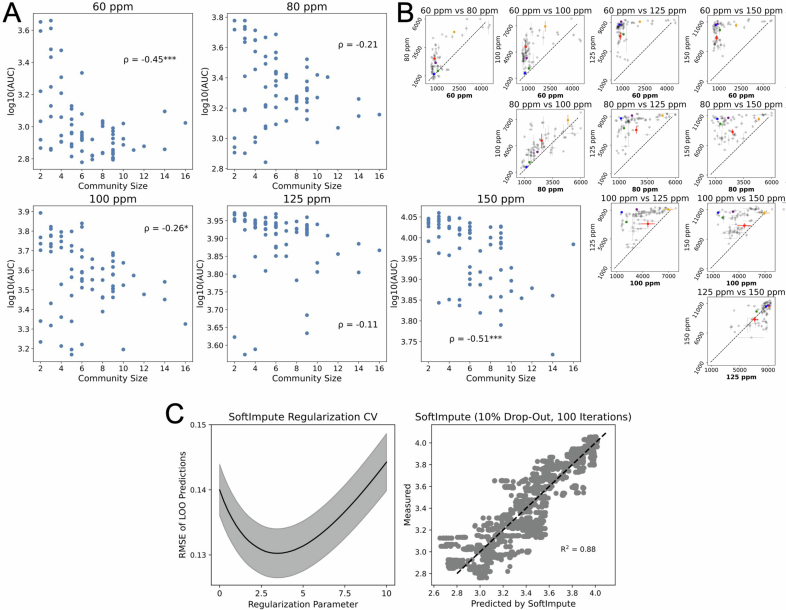
Patterns of AUC Variation Between BPA Concentration. (**A**) Scatter plots of all 70 communities showing log10AUC (yaxis, averaged across technical duplicates) versus community size (x-axis). Community size is not strongly correlated with BPA degradation in most cases. *p-value < .05 (125 ppm: 0.029), ***p-value < 0.001 (60 ppm: 1.0E-5; 150 ppm: 8E-4) calculated by the scikit-learn function pearsonr (**B**) Comparing the AUCs of our 70 communities across the five different initial concentrations. Error bars represent the standard deviation of technical duplicates. Dotted lines indicate normalized equivalent AUC. (**C**) (LEFT) Root Mean Squared Error of Leave-One-Out predictions as a function of the SoftImpute regularization parameter (see Methods). Regularization parameters were tested between 0 and 10 in increments of 0.25, and R^2^ was calculated from the complete leave-one-out predictions of the AUC matrix. The optimal regularization of 3.5 was chosen for all imputations. Shaded region indicates the standard error of all folds. (RIGHT) SoftImpute predictions from randomly dropping 10% of community AUCs. Random drops were performed 100 times, and all resulting predictions are plotted. Dotted line represents y=x. R^2^ = 0.88. Source data

We incubated each community in BPA minimal medium and monitored BPA concentrations over 72 h (see Methods). Since we expect that increasing the initial concentration renders degradation more challenging due to increasing toxicity for bacteria^29,30^, we incubated every community in five initial BPA concentrations (60, 80, 100, 125 and 150 ppm). Note that while typical environmental concentrations are often below 1 ppm^22^, contaminated BPA sites often range in BPA concentrations between ~10 and 200 ppm^38^. Figure 1d shows the degradation dynamics for all 70 communities in five initial BPA concentrations. Five representative communities are highlighted in colour across all concentrations. Consistent with our expectation, we found it increasingly harder for communities to degrade BPA as the initial concentration increases. Many communities that were able to degrade BPA at low concentrations cannot do so at higher concentrations (Mix19 and Mix70, orange and green traces, Fig. 1d), with very few communities exhibiting some level of BPA degradation at 150 ppm (Fig. 1d, bottom-centre). We also see evidence of collective effects in degradation, as the community consisting only of the 6 non-degrading isolates (Mix19) degrades BPA from 60 ppm. In addition, community size is not the dominant contributor to function in most conditions. Mix20, the community consisting of all strains, was not the fastest degrading community in any of the five initial concentrations. Further, we see strong correlation between degradation and richness in only two of the five concentrations of BPA (Extended Data Fig. 4a).

**Fig. 2:**
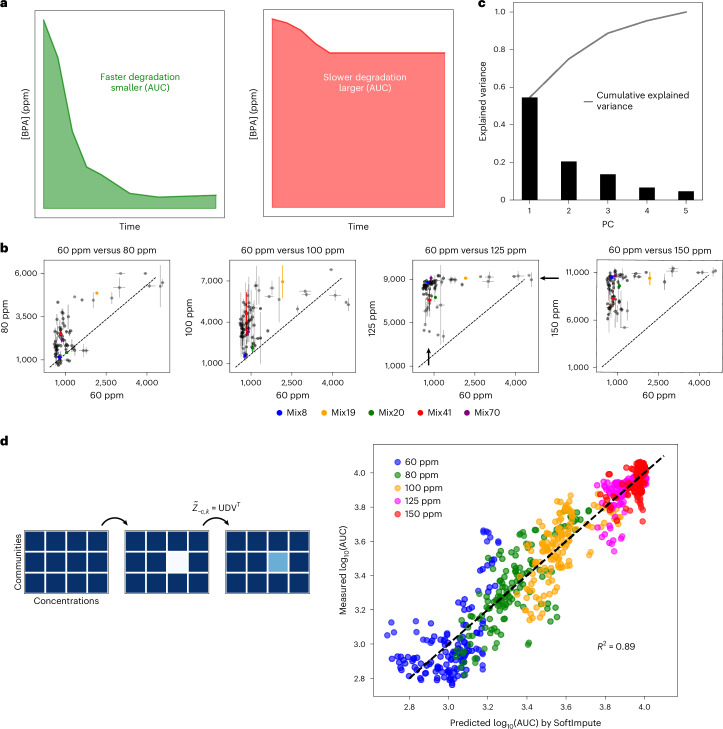
BPA degradation varies in a low-dimensional manner across concentrations. **a**, To quantify community function, we compute the AUC of BPA concentration measurements in time. A smaller AUC indicates faster and more complete degradation (left), while a higher AUC indicates the opposite (right). **b**, Comparison of the AUCs of our 70 communities between 60 ppm initial BPA and each of the other four initial concentrations (80, 100, 125, 150 ppm). Plotted points are the mean AUCs of 2 technical replicates of each community in each initial BPA concentration; error bars represent the standard deviation of AUC across both technical replicates. Dashed lines indicate normalized equivalent AUC. Two modes of variation become clearer as the difference in concentrations increases (arrows, bottom left and top right). **c**, PCA analysis shows that the first two principal components explain 79% of the variation in communities’ AUCs across concentrations. **d**, Left: schematic of SoftImpute predictions for AUC: a matrix where each row represents a community and each column an initial BPA concentration, with entries being AUC. A single entry is deleted from the matrix, and singular-value decomposition, $\widetilde{{Z}_{-c,k}}=UD{V}^{\top}$, is used to predict the deleted entry (see Methods). Right: predictions of all AUCs for all communities in all 5 initial concentrations. Predictions are performed with a log_10_ transformation of AUC. Points are coloured by the initial concentration. *R*^2^ = 0.89 for all data. Source data

To quantify community BPA degradation, we computed the area under the curve (AUC) for the BPA concentration in time, enabling us to directly compare each community across BPA concentrations (Extended Data Fig. 4b) as communities that degrade BPA rapidly have a low AUC and the converse (Fig. 2a). We observe that a community’s ability to degrade BPA from a lower initial concentration is necessary but not sufficient for that community to degrade BPA from a higher initial concentration, as no community degrades BPA faster as concentration increases (lack of points below the dashed lines, Fig. 2b). More broadly, a simple structure in community function arises as the BPA concentration increases, consisting of two axes of variation (Fig. 2b 60 ppm versus 125 ppm, black arrows). One axis comprises communities that are fast at the lower concentration but spread across AUC values at the higher concentration, and a second set of communities that are poor degraders at the higher concentration but exhibit variable AUC at lower concentrations. These patterns persist across BPA concentrations (Extended Data Fig. 4b). The relatively simple structure in the variation of AUC with BPA concentration suggested a deeper statistical interrogation of how community degradation changed with initial concentration.

### Low-dimensional variation in community function

To quantify variation in community function across BPA concentrations, we constructed a matrix of community AUCs with rows indexing composition and columns indexing initial BPA concentration. Principal component analysis on this matrix revealed two components capturing nearly 80% of the variation (Fig. 2c).

This low-dimensional structure suggests that matrix imputation via low-rank approximation could predict community function across concentrations. We log transformed and *z*-scored the AUC matrix, iteratively held out single entries, and imputed them with SoftImpute^39^ (Fig. 2d and Methods). Imputed values closely matched measurements (*R*^2^ = 0.89, *p* < 0.01), and predictions held when up to 10% of communities were held out (Extended Data Fig. 4c). Community performance at one BPA concentration can be predicted from its performance at others, suggesting simple structure in the underlying community–function landscape.

### Learning the community–function landscape for BPA degradation

The imputation method shown above enables predictions of the performance of a community across BPA concentrations so long as that community has been measured in at least one BPA concentration. However, it cannot reveal the roles of individual strains within a community in driving BPA degradation, meaning that it cannot design communities with a composition that has not already been measured.

To assess the impact of each species on the degradation of BPA, we inferred community–function landscapes. Recent work^11,13^ uses linear regression to measure the impact of strain presence and absence on community function for inferring functional landscapes^7,9^. Here we utilized the method developed in ref. ^11^. Our linear models predict log_10_(AUC) of community BPA degradation from strain presence/absence (*x*_*i*_, where *i* indexes species), and include additive terms that capture the additive impacts of individual strains (*β*_*i*_) and epistatic terms that capture the non-additive effects of species–species interactions (*γ*_*i*,*j*_, equation 1). Similar to imputation, we used log_10_(AUC) and *z*-scoring to avoid emphasizing high-AUC communities during inference (see Methods). Note that because lower AUC corresponds to faster degradation, more negative terms (*β*_*i*_,*γ*_*i*, *j*_ < 0) indicate better BPA degradation. We fit the coefficients *β*_*i*_ and *γ*_*i*, *j*_ for each of the five initial BPA concentrations independently. Since there are 136 parameters and only ~70 data points, we employed ridge regularization during inference, and the optimal regularization hyperparameter was determined by leave-one-out cross-validation. Model predictions were assessed via leave-one-out cross-validation (see Methods, Table 1, and Supplementary Fig. 1a). We find that including third-order terms does not improve model fits (Supplementary Fig. 1c).

**Table 1:** Optimal penalty terms for ridge regressions

Concentration	Value for L2 penalty term
60 ppm	82.07
80 ppm	1.10 × 10^−5^
100 ppm	133.57
125 ppm	32.41
150 ppm	16.64

**Fig. 3:**
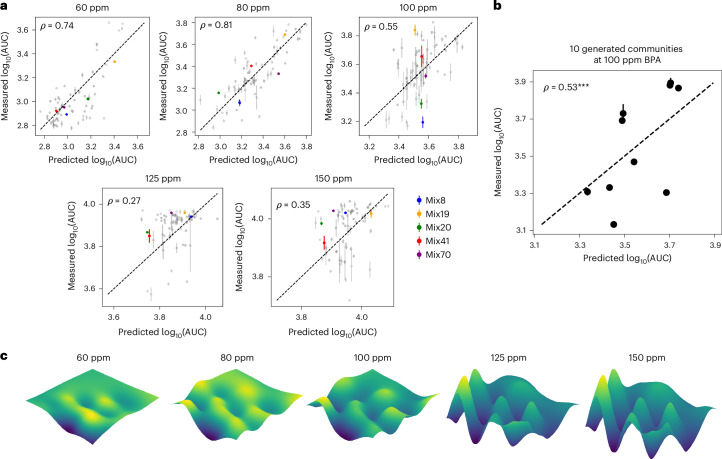
Inferring community–function landscapes enables design. **a**, For each concentration, a second-order model of the form shown in **a** is inferred. Hyperparameter tuning is performed via cross-validation. Each panel plots measured versus predicted log_10_(AUC) for each community at a single initial BPA concentration for the optimal hyperparameter. For each community, at each concentration, the model is trained on the remaining 69 communities (leave-one-out), and this model is used to predict the log_10_(AUC) for the held-out community. Both duplicates for each community composition are held out at once. This predicted log_10_(AUC) is plotted on the *x*axis and the mean measured log_10_(AUC) of two technical replicates is on the *y* axis. Vertical error bars represent the standard deviation of log_10_(AUC) across both technical replicates. The dashed lines denote perfect predictions. **b**, For the model trained in the third panel of the top row of **a**, we computationally designed 10 new communities from the model and measured their BPA degradation in a new experiment. Scatterplot shows predicted log_10_(AUC) (*x* axis) versus the mean measured log_10_(AUC) of two technical replicates (*y* xis) for these 10 communities at the initial concentration of 100 ppm. Vertical error bars represent the standard deviation of measured log_10_(AUC) across both technical replicates. *ρ* = 0.53, ***P < 0.001 (one-sided test based on 1,000 bootstrapping iterations) rejects the null hypothesis that the Pearson’s correlation coefficient (*ρ*) is ≤0. **c**, Visualization of the landscapes for each of the five initial BPA concentrations. The position in the *x*–*y* plane indicates community composition and is determined by principal components analysis on the community composition matrix using projections onto the first two components. The *z* axis then corresponds to the −log_10_(AUC) for each community, with values that are plotted after extrapolation and smoothing. The figures should not be interpreted quantitatively as the details vary depending on parameters chosen for smoothing, but the qualitative features name increasing ruggedness with BPA concentration are robust to variation in these parameters. Source data

The leave-one-out predictions are shown in Fig. 3a, where we observe generally good out-of-sample predictions, with a decrease in the quality of model predictions as initial BPA concentration increases (Fig. 3a). Optimizing these models yields a set of fitted parameters (*β*_*i*_, *γ*_*i*, *j*_) at each concentration, which collectively define the community–function landscape for BPA degradation by this library of strains.

**Extended Data Fig. 5:**
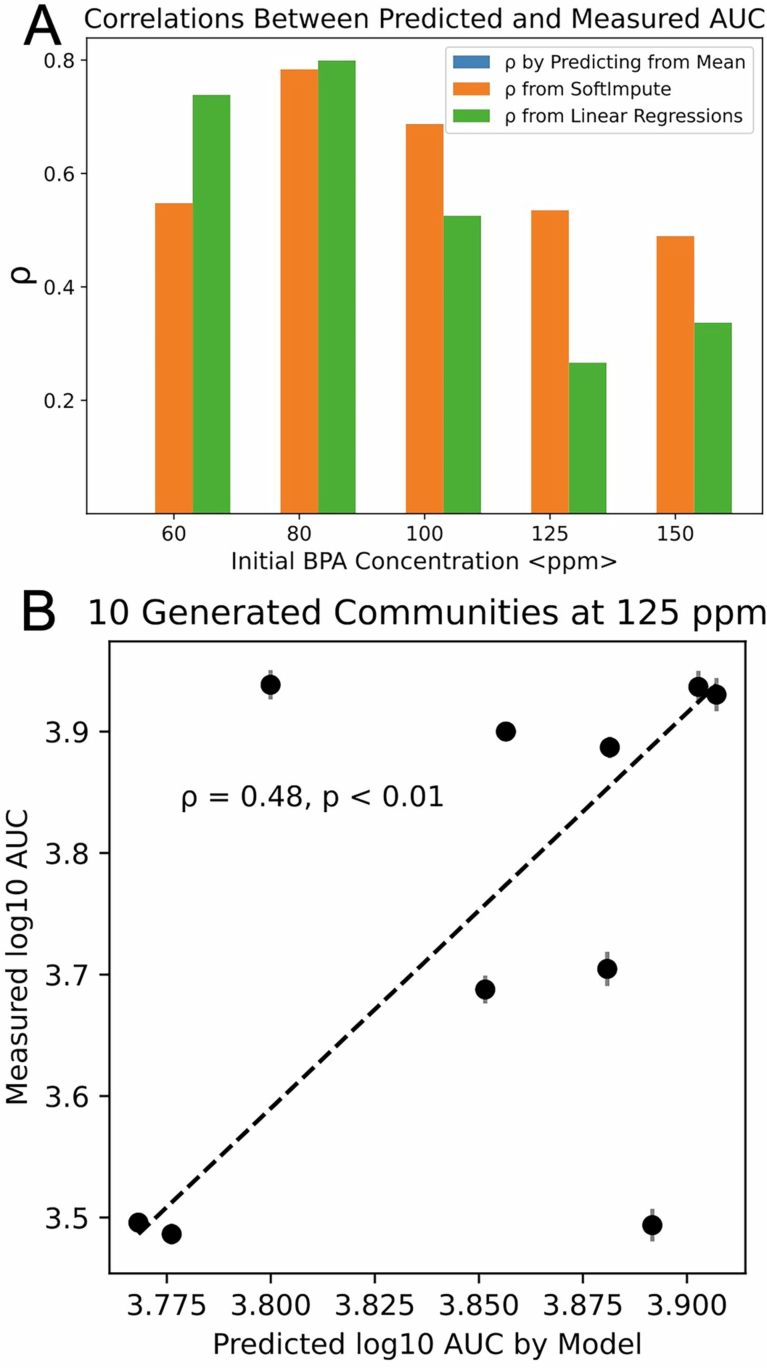
Comparison of Regression Models. (**A**) Comparing pearson’s of predicting community AUC by mean, SoftImpute, and regressions. Pearson’s correlation between measured and predicted AUC for each of the five concentrations using three models: a model that predicts the average AUC for each initial concentration (blue), SoftImpute predictions (orange, Fig. 2d), and regularized linear regressions (green, Fig. 3). Note that for the model predicting the average AUC, *ρ* = 0 for all five initial concentrations. (**B**) AUC of BPA degradation in the same ten constructed communities as Fig. 3b at an initial concentration of 125 ppm BPA. Error bars represent the standard deviation of technical replicates. Dotted line shows y=x perfect predictions. p < 0.01 from 100 bootstrapping iterations rejects the null hypothesis that the Pearson’s correlation coefficient is < 0. Source data

We find that the community–function landscapes perform just as well, if not better, than imputation (Extended Data Fig. 5a). As a null, predicting the mean log_10_(AUC) of each concentration is worse than both regressions and imputation. The relatively high performance of imputation is not surprising, as SoftImpute is given information about how each community performs at four other concentrations to predict performance at a target concentration. In contrast, regressions are only given community composition to perform predictions.

Community–function landscapes enable us to computationally design new communities from the 16 isolates with predicted high or low log_10_(AUC). Using the model fit to 100 ppm, we computationally sampled a set of previously unmeasured communities with a range of predicted performance (AUC). We then constructed these communities and measured their BPA degradation dynamics at the appropriate initial BPA concentrations. Remarkably, we find that the simple model provides good predictive power of the performance of entirely new communities (Fig. 3b and Extended Data Fig. 5b). These results establish the fidelity of our statistical approach for predicting BPA degradation as a function of community composition (for a visual representation of the community–function landscapes for each concentration of BPA, see Fig. 3c).

### Community–function landscapes reveal that toxicity drives epistasis

**Extended Data Fig. 6:**
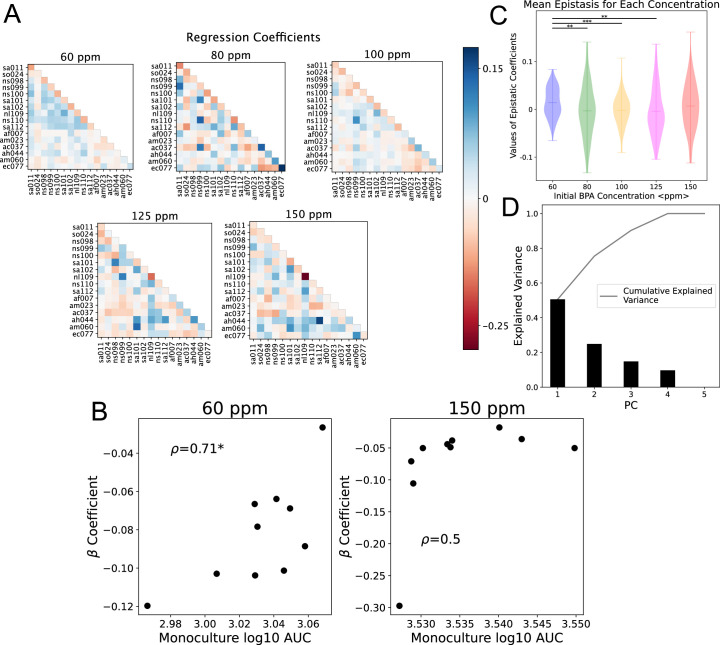
Analyzing regression coefficients to understand the functional landscape. (**A**) Heatmaps of regression coefficients from each of the five regression models (Fig. 3). Diagonal entries represent additive (*β*_*i*_) coefficients, while off-diagonal entries represent epistatic (*γ*_*i*,*j*_) coefficients. (**B**) Violin plots of epistatic coefficients (*γ*_*i*,*j*_ terms) for each initial BPA concentration. More positive values indicate more antagonistic epistatic interactions (one strain inhibiting another), while more negative values indicate more cooperative epistasis (improving BPA degradation). Horizontal lines within distributions represent the mean coefficient. **p < 0.01 (80 ppm: 0.0033; 125 ppm: 0.0090), ***p < 0.001 (100 ppm: 1.0E-4) from two-sided t-tests indicate statistically significant differences in the mean compared to 60 ppm. At 150 ppm, the mean epistasis is not significantly different from 60 ppm, but the variance of the distribution of epistatic terms is larger (Fig. 4d) (**C**) PCA on Regression Coefficients show that the first two principal components explain 75% of the variation between coefficients of the five models, while the first four PCs explain all of the variation. (**D**) For our ten degraders, monocultures were prepared in 60 ppm (left) and 150 ppm (right) BPA, and each strain’s AUC of degradation was compared to its *β* coefficient derived from the respective model. *p-value = 0.0220 as computed by the SciPy function PearsonR. Note the limited range on the x-axis in 150ppm due to little or no degradation in this condition. Source data

One way to characterize community–function landscapes is by the degree to which they are dominated by additive versus epistatic effects. Landscapes with high epistasis (many large values of *γ*_*i*, *j*_ relative to *β*_*i*_) are inherently rugged, with many local minima and maxima. Conversely, purely additive landscapes (*γ*_*i*, *j*_ = 0) are smooth and take on a Mount Fuji structure with a single optimum. Previous studies showed that epistasis is generally weak in synthetic communities^11^, leaving open the question of what drives epistasis in community–function landscapes. With this picture in mind, we compared the regression coefficients across initial BPA concentrations (Extended Data Fig. 6a).

**Fig. 4:**
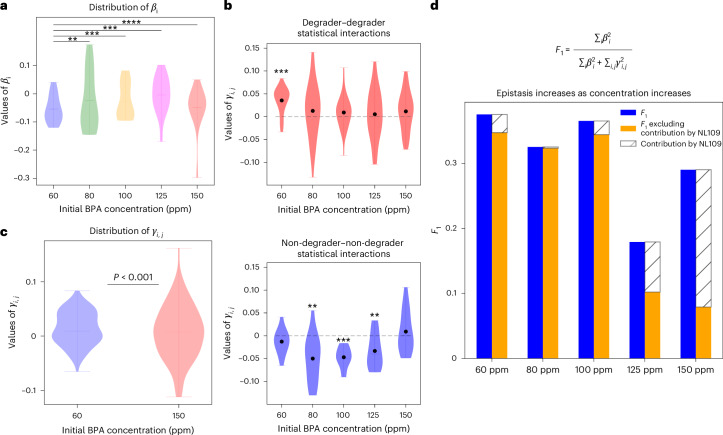
Epistasis increases as toxicity increases. **a**, Distribution of additive coefficients (*β*_*i*_) for linear models fit to each of five initial BPA concentrations (Fig. 3). Mean of coefficients rises towards zero as concentration increases. Horizontal lines within the distributions represent the mean of the coefficients. Vertical bars represent the range of coefficients, overlayed by a representation of their distribution. **P < 0.01, ***P < 0.001, ****P < 0.001 when strain NL109 is excluded (when strain NL109 is included, no longer statistically significant), all determined by two-sided 1,000 bootstrap iterations comparing the distribution of additive coefficients at 60 ppm to the additive coefficients of the other four concentrations (when compared to 60 ppm: 80 ppm BPA, *P* = 0.006; 100 ppm BPA, *P* < 0.001; 125 ppm BPA, *P* < 0.001; 150 ppm BPA without NL109, *P* < 0.001; 150 ppm BPA with NL109, *P* = 0.122). **b**, Distributions of epistatic coefficients (*γ*_*i*,*j*_) by degrader–degrader coefficients (top, red) and non-degrader–non-degrader coefficients (bottom, blue). Degrader–degrader terms are competitive (positive, slowing down degradation) at 60 ppm (*P* = 1.04 × 10^−10^ determined by a two-sided *t*-test rejecting the null hypothesis that the average terms are zero), but become neutral on average as toxicity increases. Non-degrader–non-degrader effective interactions are cooperative (negative, promoting degradation) on average for the intermediate concentrations but neutral at 60 and 150 ppm. Solid dots represent the mean of coefficients. Vertical bars represent the range of coefficients, overlayed by a representation of their distribution. **P < 0.01, ***P < 0.001 determined by two-sided *t*-tests reject the null hypothesis that the mean epistatic coefficient is 0 (80 ppm, *P* = 0.00190; 100 ppm, *P* = 1.08 × 10^−6^; 125 ppm, *P* = 0.00395). **c**, Distribution of all epistatic coefficients for models inferred at 60 and 150 ppm. Epistatic coefficients are more dispersed at 150 ppm, indicating greater epistasis at high BPA concentrations and therefore toxicity. Horizontal lines within the distributions represent the mean. Vertical bars represent the range of coefficients, overlayed by a representation of their distribution. *p* < 0.001 determined via two-sided 1,000 bootstrapping iterations rejects the null hypothesis that the two distributions are the same. **d**, Top: equation for the *F*_1_ statistic. A higher *F*_1_ statistic indicates a greater contribution by additive (*β*_*i*_) terms, thus less epistasis. Bottom: the calculated *F*_1_ statistic for each of the five models (blue). On average, *F*_1_ decreases as concentration increases, showing rising epistasis as toxicity increases. Although the *F*_1_ statistic increases at 150 ppm, this increase is due to the contribution of NL109 alone, and accounting for it maintains the pattern of decreased epistasis (orange and striped regions with and without NL109). Source data

The first property of these landscapes we note is that large, negative additive coefficients at 60 ppm generally move towards zero or positive values at concentrations >100 ppm (Fig. 4a), meaning that the average effects of individual strains on BPA degradation become less important as BPA concentration increases. We experimentally validated the inferred additive effects by measuring degradation rates for individual strains at 60 ppm initial BPA concentration. In these conditions, strains with more negative *β*_*i*_ exhibit faster degradation alone, corroborating our landscape inferences (Extended Data Fig. 6b). This trend is the first hint that the ruggedness of the landscape changes with BPA concentration.

Interestingly, the additive coefficients at 150 ppm show a single large negative coefficient, corresponding to strain NL109 (Fig. 4a, 150 ppm, and Extended Data Fig. 4b). This coefficient implies that NL109, on average, impacts BPA degradation at 150 ppm whenever it is present in a community. The additive coefficient for NL109 is slighly less negative at 125 ppm (Extended Data Fig. 6a). This supports NL109’s important role in degrading toxic concentrations of BPA despite its inability to degrade 150 ppm BPA on its own (Fig. 1c).

Next, we plotted the epistatic coefficients representing degrader–degrader and non-degrader–non-degrader statistical interactions across BPA concentrations (Fig. 4b, top). Epistatic effects between degrading strains are statistically competitive (positive *γ*_*i*, *j*_) at 60 ppm BPA and zero on average for the higher concentrations. Moreover, the spread in epistatic terms increases as the concentration rises (Fig. 4c), meaning that there are more extremely negative and positive *γ*_*i*, *j*_ as BPA concentration rises, indicating stronger epistasis.

**Extended Data Fig. 7:**
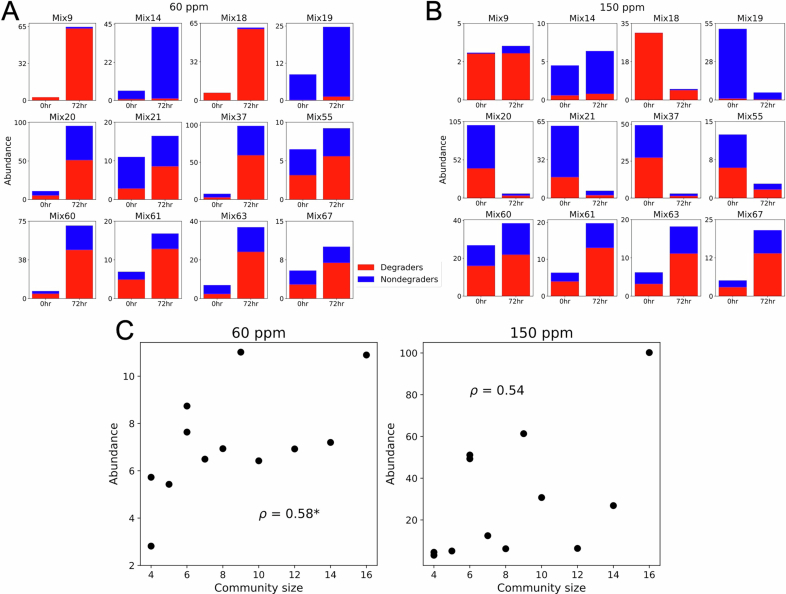
Abundances of degraders and nondegraders for 12 communities at 60 and 150 ppm BPA. Absolute abundances of degraders and nondegraders of twelve communities inoculated in 60 ppm (**A**) and 150 ppm (**B**) measured via amplicon sequencing. Absolute abundance was computed by normalizing the number of ASV reads to that of a spike-in Parabacteroides strain (see Methods). Each panel shows the abundances before (left bar) and after (right bar) BPA degradation. Red and blue sections correspond to aggregated abundances of degraders and non-degraders respectively. (**C**) Number of strains (x-axis) vs initial absolute abundance of all strains in the community (y-axis) for each of the twelve consortia at 60 ppm (left) and 150 ppm (right). *pvalue = 0.041 computed by the SciPy function PearsonR. Source data

For non-degrader–non-degrader coefficients (Fig. 4b, bottom), we observe more synergistic epistasis between strains (shown by increasingly negative mean epistasis) up to 125 ppm, before returning to zero at 150 ppm. To confirm the role of non-degrading strains in community performance, we measured absolute abundances and composition via 16S rRNA amplicon sequencing for a subset of 12 consortia that included degraders (see Methods). In all consortia, both degraders and non-degraders persisted through the end of the experiment whenever they were present, and in some cases increased in abundance. No community lost biomass in 60 ppm, although some consortia showed reduced total abundance in 150 ppm, consistent with the increased toxicity at the higher concentration (Extended Data Fig. 7). These findings further support the relevance of non-degraders in the community breakdown of BPA.

The increased epistasis can be quantified by the *F*_1_ statistic ($\sum {\beta }_{i}^{2}/(\sum {\beta }_{i}^{2}+\sum {\gamma }_{i,\,j}^{2})$) (Fig. 4d, top), which quantifies the relative contribution of additive versus epistatic terms to the landscape. Note that if *γ*_*i*, *j*_ = 0 for all *i*, *j* then *F*_1_ = 1, but as $\sum {\gamma }_{i,\,j}^{2} > > \sum {\beta }_{i}^{2}$, *F*_1_ → 0. Computing *F*_1_ statistics shows consistent metrics between 60 and 100 ppm BPA that drop considerably at 125 ppm, indicating that epistatic terms dominate as the concentration increases (Fig. 4d, bottom). The increase in *F*_1_ at 150 ppm is solely explained by the large *β*_*i*_ term corresponding to NL109 (discussed above). Removing the contribution from this strain reveals increasing epistasis at 150 ppm. Consistent with this finding, community size was significantly correlated with BPA degradation in both 60 and 150 ppm (Extended Data Fig. 4a), where additive effects dominate at 60 ppm and strain NL109 exhibits a large effect at 150 ppm.

The conclusion that epistasis rises with BPA concentration strongly suggests that BPA degradation becomes a more collective, community-level process as toxicity increases (Extended Data Fig. 6c). This is confirmed by the observation that no individual strain is capable of degrading BPA at 150 ppm, despite their ability to do so at 60 ppm (Fig. 1c).

Finally, the observation that epistasis increases with concentration and additive coefficients become less important raises a question regarding the success of the imputation methods shown in Fig. 2d due to the low-dimensional structure of variation in AUC across BPA concentrations. It is unclear how this low dimensionality manifests in our inferred community–function landscapes because models for each concentration were inferred independently. We suspected that the variation in community–function landscapes between concentrations should also be low dimensional. To test this idea, we performed principal components analysis (PCA) on the matrix of regression coefficients across BPA concentrations (Methods) and found that 75% of the variance in coefficients is explained by the first two components (Extended Data Fig. 6d). The result suggests that the community–function landscape changes in a simple manner as the concentration of BPA rises.

The low-dimensional variation in landscape coefficients suggests that we might be able to infer landscapes across BPA concentrations in a single regression, assuming low-dimensional variation in community–function landscape coefficients. We developed a regularized optimization procedure to accomplish this and found that the variation in AUC across BPA concentrations enabled such an approach (Supplementary Fig. 4 and Methods).

### Functional landscape enables design for remediation in soils

Our in vitro experiments used minimal medium with BPA as the sole carbon source and no competing microbiome. Remediation contexts—the gut^14,15,40^ or soils^41,42^—require engraftment of inoculated microbes against an existing microbiome.

Engraftment is proposed to succeed when the inoculum occupies a niche weakly held by the native microbiome^18^. Since BPA degradation is not readily performed by most bacterial taxa^43^, we tested whether our in vitro-designed consortia could degrade BPA in soils.

**Fig. 5:**
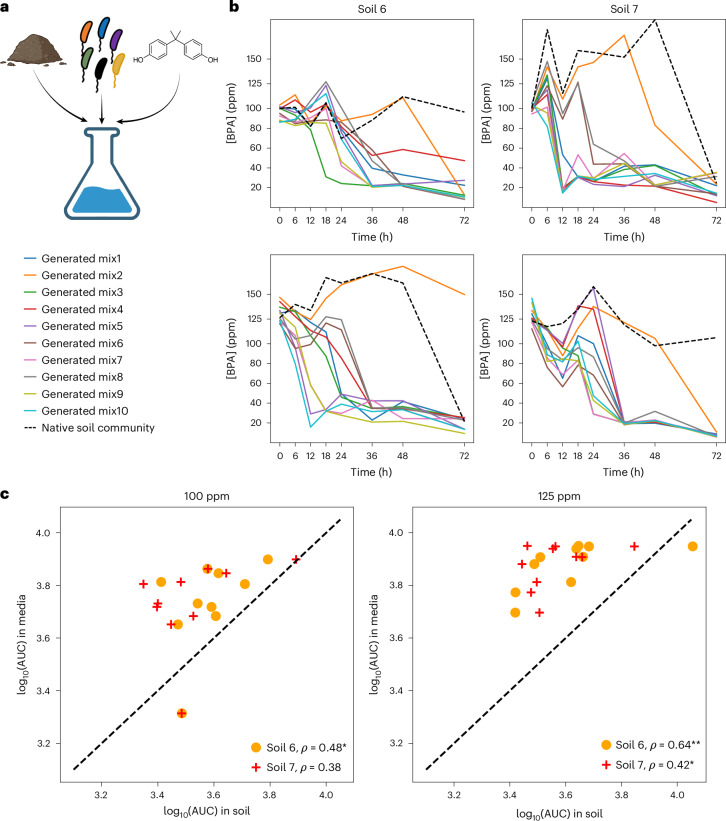
Designing for BPA remediation in soils. **a**, Schematic for BPA remediation protocol. Soil was combined with BPA-contaminated water to form a slurry. Contaminated slurries were inoculated with synthetic communities, and BPA degradation was assayed (see Methods). **b**, BPA degradation curves for the 10 synthetic communities in two soils and two initial BPA concentrations: 100 ppm (top) and 125 ppm (bottom). The black dashed line shows BPA degradation by the native soil microbiome without an added synthetic community. **c**, Scatterplots showing the 10 communities’ AUC (Fig. 2) in soil (*x* axis) and in vitro (*y* axis) for two soils and two initial BPA concentrations. Dashed lines denote equality of AUCs. *ρ* in the legend denotes Pearson’s correlation between in vitro and in soil degradation of BPA. *P < 0.05, **P < 0.01 (one-sided test based on 1,000 bootstrapping resamplings with replacement) reject the null hypothesis that the Pearson’s correlation coefficient is <0 (100 ppm: Soil 6 *p* = 0.034, Soil 7 *p* = 0.067 (not significant); 125 ppm: Soil 6 *P* < 0.001, Soil 7 *P* = 0.024). Panel **a** created with BioRender.com. Source data

**Extended Data Fig. 8:**
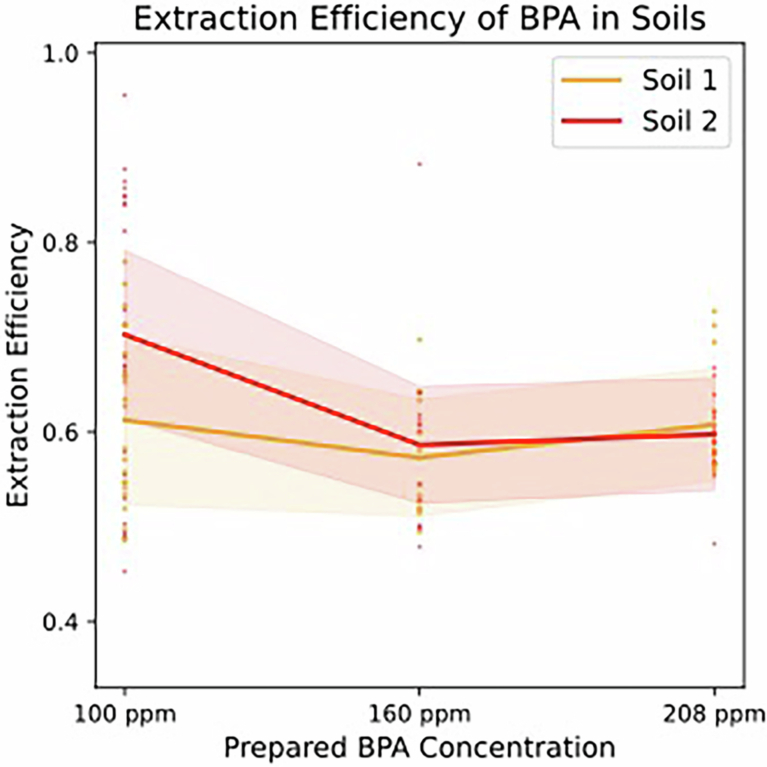
BPA extraction efficiency from soil slurries. Extraction efficiency of BPA from both soil slurries from three initial BPA concentrations. Solid colored lines represent the mean extraction efficiency for each soil at each concentration, and shaded regions denote one standard deviation from the mean. Number of replicates varied between 20 and 40 for each condition. For extraction protocol see Methods. Source data

We prepared BPA-contaminated slurries from two soils at two BPA concentrations (100 and 125 ppm), inoculated each with the 10 in vitro-designed communities (Fig. 3b), and measured BPA in time via sampling (Fig. 5a, Methods and Extended Data Fig. 8). Uninoculated slurries controlled for degradation by the native soil microbiome.

Inoculation increased BPA degradation, and in some cases the native microbiome could not degrade BPA on its own (Soil 1, 100 ppm; Soil 2, 125 ppm). Degradation dynamics depended on which community was inoculated, showing that composition affects BPA remediation even in the presence of a complex indigenous microbiome.

Given that our landscape models enable rational community design (Fig. 3), we asked whether in vitro performance predicts performance in soils. We compared each community’s AUC in vitro with its AUC in soils. We do not expect a 1:1 relationship given the challenges of BPA extraction from soil (Methods), but the AUCs correlate for both soils (Fig. 5). Thus, landscapes learned in vitro are informative of community performance in soils.

## Discussion

Functional landscapes are most easily inferred when they are relatively smooth with limited epistasis ^11^ and when limited to a stable environment^11,12,14^. Indeed, most of the previous work on landscapes falls into both categories. The BPA degradation landscape presented here offers a different picture: the landscape shows smooth, low ruggedness at low BPA concentrations, changing to high ruggedness at high BPA levels (Fig. 4 and Extended Data Fig. 6). However, the higher concentrations were more difficult to fit than the lower concentrations (Fig. 3a). We attribute this to the sparse sampling of 70 communities (out of a possible ~65,000) in a regime where epistatic terms dominate (Fig. 4d). Despite this difficulty, our work serves as a proof of principle for analysing community function landscapes across environmental gradients.

We showed that community composition affects BPA degradability in a non-trivial manner. At low BPA concentrations, the degradation is primarily driven by the presence of degraders (Fig. 4a). As the concentration increases, these interactions become required for BPA degradation to occur. Exploiting these statistical interactions enabled not only accurate community design in laboratory conditions, but also engraftment in soil conditions akin to natural contexts^18,19,44,45^. Our success paves the way for simplifying similar endeavours for other bioremediation targets.

Although our work shows translational potential for bioremediation efforts, it remains a simplification of natural systems. Natural microbiomes are extremely diverse and often compete with inoculated consortia for resources or disrupt their metabolic pathways^46,47^. In addition, heterogeneous abiotic factors such as pH and nutrient availability dramatically alter natural microbial function^48^. These dynamics, which are difficult to capture in controlled systems, may substantially affect the engraftment and functional activity of synthetic consortia. The niche specialization theory^18^ qualitatively explains the success of soil inoculations in our work, but may face challenges in natural contexts.

While niche specialization probably enabled BPA bioremediation in a soil context, generalization into polluted environments remains complicated. Often, polluted environments harbour multiple contaminants, which may interact with each other chemically or metabolically^49^. For example, polycyclic aromatic hydrocarbon (PAH) pollution affects the ability of microbes to degrade heavy metals by altering usable chemical pathways^50^. Biodegradation in these environments requires accounting for these effects both experimentally and computationally.

## Methods

### BPA media

BPA medium was prepared using 1× M9 minimal medium with BPA as the sole carbon source (Supplementary Table 2). BPA was dissolved in 1× M9 medium and autoclaved for 30 min at 121 °C. The final concentration of the medium was verified by the assay described below before use.

### Soil sampling and enrichment

Five sites were identified as being potentially BPA contaminated—two directly adjacent to BPA-producing factories, and three directly downstream from these factories (Supplementary Table 1, sites 1–5). Approximately 2 kg of topsoil from each site was collected and refrigerated at 4 °C for ~1 month before use.

Soil enrichments were performed in 30 ppm BPA M9 media. Enrichments were performed in 250 ml Erlenmeyer flasks, containing 3 g of soil in 60 ml of BPA media. Cycloheximide (400 μg ml^−1^) and nystatin (40 μg ml^−1^) were added to inhibit eukaryotic growth. The solution was vortexed, and a 40-μl sample was taken for initial BPA concentration measurement. Flasks were incubated aerobically at 30 °C, with shaking at 220 rpm.

BPA concentration was measured daily. Whenever the measured BPA concentration fell below half of the starting concentration, the sample would be diluted into fresh BPA media adjusted to a final volume of 60 ml, for up to three rounds of serial dilution. The dilution factor increased with each round: the first was 1:16 (3.75-ml sample with 56.25-ml BPA media), then 1:32, then 1:64. The purpose of dilutions was to select for microbes that either degrade BPA or co-exist with degraders in the presence of BPA.

From soils that degraded BPA after the third dilution, samples were diluted in PBS to a 10^−5^ dilution and plated on R2A plates (R2 broth, Research Products, R24260; agarose, Fisher Scientific, BP1356). Plates were incubated at 30 °C to encourage growth. Colonies were picked and then streaked to purity, after which isolates were mixed 1:1 with 50% glycerol and stored at −80 °C.

### Individual strains

Sixteen strains were isolated and identified from soil enrichments. Ten of these strains degraded 30 ppm BPA on their own, while six did not. Our BPA-degrading strains came from two soil samples, while our non-degraders came from the other three samples.

During the revision process of this manuscript, whole-genome sequencing was performed through Plasmidsaurus using Oxford Nanopore Technology to isolate the 16S sequences of each strain and determine isolate purity (Extended Data Table 1). Overnight cultures were prepared from frozen glycerol stocks of each of our colonies for 48 h, and DNA was extracted via DNeasy PowerSoil Pro kit (QIAGEN, 47014) and shipped immediately for sequencing.

We note that the impurities detected impacted neither the reproducibility of our experiments nor the interpretation of the associated landscapes. However, they do preclude making statements about the physiology or metabolism of these four isolates in our library from our community-level data.

Using the 16S sequence of each strain obtained from whole-genome sequencing (above), strain taxonomy was identified through NCBI BLAST^51^. The EMBL-EBI Job Dispatcher^52^ was used to align sequences via Muscle and construct the phylogenetic tree (Fig. 1b), and the Interactive Tree of Life^53^ was used to visualize and edit the tree.

### Measuring CO_2_

CO_2_ production in our ten degrading isolates by BPA degradation was measured using a Microresp assay (Extended Data Fig. 3). A monoculture of each strain was prepared as described in Methods (‘Synthetic community experiments’). Four technical replicates were prepared: two in 60 ppm BPA media, and two in M9 media with no carbon source. Of each replicate, 700 μl was transferred into a 96-deep-well plate, which was then sealed with a pH-sensitive dye in an agarose gel. Respiration of CO_2_ reduces the pH of the dye, thus changing its colour (see ref. ^54^ for more information on the setup of the Microresp system). The plate was incubated at 30 °C with shaking at 900 rpm for 3 days, after which CO_2_ respiration was measured by an absorbance reading of the dyes.

### BPA assay

BPA concentrations were measured using a modified version of the phenol colorimetric assay^55^ optimized for high-throughput use in a 96-well plate. Under basic pH conditions, 4-aminoantipyrine (4-AAP) reacts with BPA in an electrophilic substitution reaction, which can then be oxidized by potassium ferricyanide to produce a red dye that absorbs at 506 nm in proportion to the initial BPA concentration in the sample. Reagents were added in the following order: 120 μl 0.25 M sodium bicarbonate (Sigma-Aldrich, S6014) to serve as a basic buffer, 40 μl of a BPA-containing sample, 40 μl 20.8 mM 4-AAP (Sigma-Aldrich, A4382) and 40 μl 83.4 mM potassium ferricyanide (Arcos Organics, 424125000). The plate was incubated at room temperature with shaking at 750 rpm for 45 s, and then incubated statically for 10 min. Afterwards, the absorbance of each sample was read at 506 nm. A standard curve was made from solutions of known BPA concentration in BPA minimal media as described above for every timepoint in every experiment, and each 96-well plate was prepared with its own standard curve. The standard curves had a slope of 133.36 ± 13.14 and an intercept of −0.88 ± 3.1 (Extended Data Fig. 1).

### Synthetic community experiments

The initial set of 70 communities had a mean richness of 6 ± 3 (s.d.) and included a community of all 16 strains, a community of all 10 degraders, and a community of all 6 non-degraders. The remaining 67 communities were assembled randomly.

To prepare our strains for mixing into synthetic communities, each of the strains was sampled from its frozen stock and grown anaerobically in 5 ml Tryptic Soy Broth (TSB) media for 44 h in tightly closed Falcon tubes, incubating at 30 °C and 220 rpm. After incubation, samples were centrifuged at 3,100 *g* for 15 min and the supernatant was pipetted out. Pellets from each sample were resuspended in 1 ml M9 media as a wash step, and centrifuged again at 3,100 *g* for 8 min, after which the supernatant was pipetted out again. Finally, pellets were suspended in 2 ml of BPA media, and samples were ready for community assembly.

All communities were mixed and incubated in Fisher 48-deep-well plates (1162B95), with one well housing one community. Each well was filled with 2 ml of M9-BPA medium, and each isolate was added at an initial nominal OD of 0.02 irrespective of the community richness. The initial OD of each strain was kept consistent to allow for estimation of a strain’s average impact on degradation. If the initial OD was changed in proportion to community size (to have fixed total OD in all communities, for example), the impact of every strain would be dependent on community size. As such, this protocol does not enable elucidating the dependence of lag phase or rate on inoculum size.

All wells were mixed thoroughly by pipetting up and down repeatedly, and then incubated at 30 °C in a Fisher Scientific benchtop microplate shaker (02-217-757), shaking at 900 rpm, covered by a breathe-easy membrane (Sigma-Aldrich, Z380059). Every plate also contained at least one well with no bacteria added to it (as an evaporation control) and one well with TSB medium (as a contamination/splashing control). Within each BPA concentration (and soil condition for soil spike-in experiments), every community was prepared in technical duplicates, where both instances of each community were prepared from the same overnight stocks of individual strains. Different overnight stocks were used for each experimental condition.

Samples were taken at time points 0, 6, 12, 18, 24, 36, 48 and 72 h, and the BPA concentration was measured for each sample at each time point as previously described. Positive controls ensured that degradation dynamics across different batches of experiments were comparable, while negative controls detected contamination and also served as evaporation controls.

BPA degradation was quantified by computing the AUC (Fig. 2a). We chose this metric over explicit modelling of BPA degradation dynamics because the degradation patterns we observed were complex, with differing timescales and plateauing mid-way through degradation.

### Imputation to predict AUCs across concentrations of BPA

In Fig. 2, we predicted community AUCs via matrix imputation. Imputing entries from our AUC matrix *Z* (Fig. 2) was done via the R package SoftImpute. Every entry of *Z* is denoted *z*_*c*,*k*_, which is the log_10_(AUC) of community *c* in concentration *k*. AUC was log transformed to put all data on relatively the same scale (ranging from ~2.8 to 4.1). We iteratively dropped out every entry of the matrix (along with all technical replicates) to achieve *Z*_−*c*,*k*_ with community *c* in concentration *k* dropped out. We then processed *Z*_−*c*,*k*_ to be $\widetilde{{Z}_{-c,k}}$, where every column *k* was *z*-scored (such that within each concentration the response variable had a mean of 0 and a standard deviation of 1), and fit SoftImpute using the singular-value decomposition: $\widetilde{{Z}_{-c,k}}=UD{V}^{\top }$, where *D* is soft-thresholded (D^*^) to minimize its nuclear norm. The *Z* matrix was reconstructed by D^*^ as Z^*^ = UD^*^V^⊤^, where ${z}_{c,k}^{* }$ is the imputed value of the dropped entry. Leave-one-out cross-validation was done to choose the soft-thresholding regularization parameter over a range of 40 values between 0 and 10, which maximizes the total *R*^2^ of predictions (Extended Data Fig. 4c). For all fits, the resulting D^*^ was rank 2.

We also performed a similar imputation by randomly dropping out 10% of the entire matrix (accounting for technical replicates), repeated over 100 iterations of 10% dropout (Extended Data Fig. 4c).

### Linear regressions

Regressions were performed using the Python scikit-learn package. Ridge was chosen over LASSO because the *L*_2_ penalty decreases all coefficient values to avoid overfitting, while *L*_1_ regularization performs variable selection which can be problematic when regressors are correlated^56^. For each concentration *k*, models were fit of the form

1 $${y}_{c}^{(k)}={\beta }_{0}^{(k)}+\mathop{\sum }\limits_{i}{\beta }_{i}^{(k)}{X}_{i}+\mathop{\sum }\limits_{i,\,j}{\gamma }_{ij}^{(k)}{x}_{i}{x}_{j},$$

where ${y}_{c}^{(k)}$ is the *z*-scored log_10_(AUC) of community *c* in concentration *k* (similar to our imputation method above, ${y}_{c}^{(k)}$ is *z*-scored within each model for concentration *k*), ${\beta }_{0}^{(k)}$ is the intercept that is fit from the data for concentration *k*, *X*_*i*_ represents the presence and absence of strain *i* in the community *c*, denoted in +1/−1 notation to simplify interpreting coefficients ^11,57,58^, ${\beta }_{i}^{(k)}$ is the additive coefficient for stain *i* in the model fit from concentration *k*, and ${\gamma }_{i,\,j}^{(k)}$ is the pairwise coefficient for strains *i* and *j* together in the community *c* in the model fit from concentration *k*. The objective function for each model was

2 $$\begin{array}{l}\mathop{\mathrm{argmin}}\limits_{\beta ,\gamma }\left\Vert {w}_{c}^{(k)}\left({y}_{c}^{(k)}-\left({\beta }_{0}^{(k)}+\mathop{\sum }\limits_{i}{\beta }_{i}^{(k)}{X}_{i}+\mathop{\sum }\limits_{i,\,j}{\gamma }_{ij}^{(k)}{x}_{i}{x}_{j}\right)\right)\right\Vert^{2}\\ \,\,\,\,\,\,\,\,\,\,\,\,\,\,\,\,\,\,\,\,\,\,\,\,\,\,\,\,\,\,\,\,\,\,\,\,\,\,\,\,\,\,\,\,\,+\lambda \left(\mathop{\sum }\limits_{i}{\left({\beta }_{i}^{(k)}\right)}^{2}+\mathop{\sum }\limits_{i,\,j}{\left({\gamma }_{ij}^{(k)}\right)}^{2}\right)\end{array}$$

where ${w}_{c}^{(k)}$ is the weight applied to community *c* in the model for concentration *k*, and *λ* is the *L*_2_ penalty term. Weights were determined through a Gaussian kernel density estimate of community AUCs using scikit-learn. For each ${y}_{c}^{(k)}$, the estimator computes

3 $${\rho }_{k}\left({y}_{c}^{(k)}\right)=\mathop{\sum }\limits_{i=1}^{N}\frac{1}{h\sqrt{2\pi }}\,\exp \,\left(-\frac{{\left({y}_{c}^{(k)}-{y}_{i}^{(k)}\right)}^{2}}{2{h}^{2}}\right)$$

where ${y}_{i}^{(k)}$ represents all measured *y*^(*k*)^, and *h* represents the bandwidth of the curves. For each concentration, a grid search of *h* between 10^−3^ and 1 was performed to find the bandwidth that maximized the log-likelihood of the estimated distribution of all ${y}_{c}^{(k)}$. For all other parameters, the default scikit-learn settings were used. The final weight ${w}_{c}^{(k)}$ was set to be $\frac{1}{{\rho }_{k}({y}_{c}^{(k)})}$ so that a community whose AUC is more ‘rare’ within concentration *k* is given greater weight in the regression.

The optimal *λ* was chosen through leave-one-out cross-validation over a range of 10,000 values between 10^−5^ and 10^10^ and finding the value that minimized the objective function above (similar to our imputation method above, *z*-scoring of ${y}_{c}^{(k)}$ was only performed after excluding the validation community). Once the optimal *λ* was identified, the predicted value for each community within each concentration was plotted against the measured value (Fig. 3a). A separate model was fit to all *y*^(*k*)^ within each concentration *k*, each with its own optimal *λ* (Table 1). The cross-validation for the model fit to data from 80 ppm BPA yielded the smallest tested value for *λ* (near 0), in stark contrast to the other four models. The strong model fit (Fig. 3a) indicates the general ease of fitting the model to these data compared to data from the other four initial concentrations.

### Randomly shuffling the response variables of the linear regressions

As a way to validate our linear regressions against a null model (see Supplementary Discussion), within each initial BPA concentration *k*, we de-coupled ${y}_{c}^{k}$ from community composition *c* by randomly shuffling the order of the response variable ${y}_{c}^{k}$. After random shuffling, models were fit in the same way as described above. The weighting scheme was not changed; therefore, every ${y}_{c}^{k}$ retained its weighting ${w}_{c}^{k}$.

### PCA analysis

PCA analysis was performed on our matrix of regression coefficients (Extended Data Fig. 6a) to determine the variation in community function landscapes. For each concentration *k*, the column of our matrix becomes $[{\beta }_{0}^{(k)},\,{\beta }_{1}^{(k)},\,{\beta }_{2}^{(k)},\,\ldots ,\,{\beta }_{16}^{(k)},\,{\gamma }_{1,2}^{(k)},\,{\gamma }_{1,3}^{(k)},\,\ldots ,\,{\gamma }_{16,15}^{(k)}]$. The coefficients were scaled by subtracting the mean and normalizing by the standard deviation within each concentration before being subjected to PCA transformation.

### Computing alternative response variables

To determine whether the epistasis results discussed in Fig. 4 are general properties of the BPA degradation landscape, we defined alternative response variables to serve as proxies for BPA degradation efficiency.

To define a proxy for a community-specific degradation rate, we estimated a mean temporal slope over the interval of active degradation. To identify this interval, we took each initial BPA concentration *k* and computed the variance of BPA concentrations across communities as a function of time. The onset of degradation was operationally defined as the first time point at which cross-community variance increased by more than 40% relative to its initial value, indicating divergence in degradation dynamics. The endpoint was taken as the time of maximal variance, corresponding to peak separation among community trajectories. For each community *c*, the degradation rate was then defined as the slope

4 $${s}_{c}^{k}=\frac{\left|{[\mathrm{BPA}]}_{c,{t}_{0}}-{[\mathrm{BPA}]}_{c,{t}_{\max }}\right|}{{t}_{\max }-{t}_{0}},$$

where *t*_0_ and *t*_max_ denote the start and end of the active degradation window, respectively.

As a complementary summary statistic, we defined a variance-based metric for each community as the BPA concentration measured at the time of maximal cross-community variance. Specifically, for concentration *k*, the response variable was

5 $${v}_{c}^{k}={[\mathrm{BPA}]}_{c,{t}_{\max }},$$

where ${t}_{\max }$ is the time point at which the variance across communities is maximal.

For each of these two alternative response variables (${s}_{c}^{k}$ and ${v}_{c}^{k}$), we fit regression models and analysed epistasis exactly as described above.

### In-silico sampling of landscapes to understand the effect of model fit performance on inferred epistasis

To design synthetic landscapes, we considered a system of *N* = 10 binary variables representing strains, denoted as *x*_*i*_ ∈ {−1,1} for *i* ∈ {1,…,*N*}. ${x}_{i}^{c}$ defines the presence or absence of strain *i* in community *c*. For each community *c*, we defined the corresponding functional output *y*_*c*_ using a second-order model of the same form as equation 1:

6 $${y}_{c}=\mathop{\sum }\limits_{i=1}^{N}{\beta }_{i}{x}_{i}^{(c)}+\mathop{\sum }\limits_{i=1}^{N}\mathop{\sum }\limits_{j > i}^{N}{\gamma }_{i,\,j}{x}_{i}^{(c)}{x}_{j}^{(c)}+{\eta }_{c}$$

where *y*_*c*_ is the functional output of community *c*, *β*_*i*_ and *γ*_*i*, *j*_ are additive and pairwise coefficients, respectively (see above), and ${\eta }_{c} \sim {\mathcal{N}}(0,{\sigma }^{2})$ is independent Gaussian noise with variance *σ*^2^.

We generated a family of synthetic models and controlled the true *F*_1_ statistic of the model to be between 0.1 and 1, in increments of 0.1. This was achieved by varying the proportion of additive (*β*_*i*_) versus epistatic (*γ*_*i*, *j*_) coefficients.

For each model *f* (corresponding to a fixed *F*_1_ statistic), we generated *S* = 60 communities, denoted as *X*^*s*^. We then sampled from *X*^*s*^ a number of communities $s{\prime}$, varying from $s{\prime} =5$ to $s{\prime} =55$. For each sampling size $s{\prime}$, we generated *R* = 100 independent random subsamplings. Each subsampling was denoted ${X}_{r}^{s{\prime} }{| }_{r=1}^{R}$. We also added a sample for all 60 communities, that is, $s{\prime} =60$. We then computed functional outputs for the sampled communities, ${y}_{c}^{s{\prime} ,r}=f({x}_{c}^{s{\prime} ,r})$ for each community ${c}^{s{\prime} ,r}\in {X}_{r}^{s{\prime} }$ (for now, *σ*^2^ = 0 in *f*, thus *η*_*c*_ = 0 for all communities *c*). From the complete dataset $\{{X}_{r}^{s{\prime} },{y}_{c}^{s{\prime} ,r}\}$, we fit a regression model in the same way as in the main analysis (see Methods) and inferred coefficients $\widehat{{\beta }_{i}}$ and ${\widehat{\gamma }}_{i,\,j}$. We used these inferred coefficients to calculate the inferred *F*_1_ statistic. This was repeated for all sample sizes $s{\prime}$ and for all 100 random subsamplings of ${X}^{s{\prime} }$, such that for each *F*_1_ value of *f*, a distribution was generated for each inferred *F*_1_ statistic for each sample size $s{\prime}$, denoted ${\widehat{F}}_{1}^{s{\prime} ,r}$. The mean of all 100 subsamplings was reported as a final ${\widehat{F}}_{1}^{s{\prime} }$.

### Abundance of consortia at community endpoint

We performed 16S amplicon sequencing on 12 of our communities (Mixes 9, 14, 18, 19, 20, 21, 37, 55, 60, 61, 63 and 67) inoculated at initial BPA concentrations of 60 ppm and 150 ppm. These 12 communities were chosen due to the wide representation of community sizes and the relative compositions of degraders and non-degraders. All 12 communities were prepared in technical triplicates, and 400-μl samples were taken at the start (*t* = 0 h) and end (*t* = 72 h) points. This led to a total of 144 amplicon sequencing measurements. From the 400-μl samples, DNA was extracted using the DNeasy 96 PowerSoil Pro kit (QIAGEN, 47017), following manufacturer protocol, and then stored at −20 °C. DNA Library preparation was performed using the 16S metagenomic sequencing library preparation protocol with a 2-stage PCR workflow (Illumina). A known quantity of genomic DNA from *Parabacteroides* sp. TM425 was added to each sample before extraction to estimate the absolute abundance of consortia. The V3–V4 region was amplified using the forward primer 341-b-S-17 (CCTACGGGNGGCWGCAG) and the reverse primer 785-a-A-21 (GACTACHVGGGTATCTAATCC)^59^. PCR products were confirmed by gel electrophoresis and purified. Sequences were obtained on the Illumina MiSeq platform in a 2 × 300-bp paired-end run using the MiSeq Reagent Kit v.3 (Illumina). Raw amplicon sequences were processed using the DADA2 pipeline (v.1.36) in R (v.4.5.1) to filter, denoise, merge and remove chimaeric reads. Taxonomy assignment was performed at the genus level with DADA2 using the SILVA database v.138.1

### Fitting the low-rank regressor

The low-rank regressor is an attempt to combine the regressions of all five BPA concentrations to infer all sets of coefficients in a low-dimensional manner. It assumes the relationship

7 $$Z\approx X\Theta Y,\,\,\mathrm{for\; an\; approximately\; low}\mbox{-}\mathrm{rank\; matrix}\,\Theta \in {{\mathbb{R}}}^{p\times r},$$

where $Z\in {{\mathbb{R}}}^{{n}_{\mathrm{comm}}\times {n}_{\mathrm{BPA}}}$ is the matrix of community AUCs under all available *n*_BPA_ concentrations, $X\in {\left\{\pm 1\right\}}^{{n}_{{\rm{comm}}}\times p}$ is the community composition matrix and $Y\in {{\mathbb{R}}}^{r\times {n}_{\mathrm{BPA}}}$ is a polynomial basis matrix, with each row comprising monomials evaluated at the available BPA concentrations:

8 $$Y=\left[\begin{array}{cccc}1 & 1 & \ldots & 1\\ {\phi }_{1}({{\mathsf{PPM}}}_{1}) & {\phi }_{1}({{\mathsf{PPM}}}_{2}) & \ldots & {\phi }_{1}({{\mathsf{PPM}}}_{{n}_{\mathrm{BPA}}})\\ {\phi }_{2}({{\mathsf{PPM}}}_{1}) & {\phi }_{2}({{\mathsf{PPM}}}_{2}) & \ldots & {\phi }_{2}({{\mathsf{PPM}}}_{{n}_{\mathrm{BPA}}})\\ \vdots & & & \vdots \\ {\phi }_{r}({{\mathsf{PPM}}}_{1}) & {\phi }_{r}({{\mathsf{PPM}}}_{2}) & \ldots & {\phi }_{r}({{\mathsf{PPM}}}_{{n}_{\mathrm{BPA}}})\end{array}\right].$$

Here, *r* is the degree of the polynomial, *ϕ*_1_, …, *ϕ*_*r*_ are the elements in the polynomial basis, and ${{\mathsf{PPM}}}_{j}$ denotes the *j*^th^ BPA concentration. In our experiments, we chose $\left\{{\phi }_{j}\right\}$ to be the monomials, with *ϕ*_*k*_(*x*) ≔ *x*^*k*^; however, alternative choices may be able to provide better fits. Under this formalism, the product $\Theta Y\in {{\mathbb{R}}}^{p\times {n}_{{\rm{BPA}}}}$ is the matrix of coefficients for all observations and satisfies rank(*ΘY*) ≤ *r* by construction.

We calculated an estimate $\widehat{\Theta }$ from data by solving the following optimization problem:

9 $$\widehat{\Theta }:=\mathop{\mathrm{argmin}}\limits_{\Theta }{\left\Vert W\odot (Z-X\Theta Y)\right\Vert }_{{\rm{F}}}^{2}+{\lambda }_{1}{\left\Vert \Theta \right\Vert }_{* }+{\lambda }_{2}{\left\Vert \Theta \right\Vert }_{{\rm{F}}}^{2},$$

where $W\in {{\mathbb{R}}}_{+}^{{n}_{\mathrm{comm}}\times {n}_{\mathrm{BPA}}}$ is a non-negative weighting matrix (see Methods for our weighting scheme) and:

- ⊙ denotes the elementwise (Hadamard) product;
- ${\left\Vert \Theta \right\Vert }_{* }:={\sum }_{i=1}^{r}{\sigma }_{i}(\Theta )$ is the ‘nuclear’ norm, equal to the sum of all singular values of *Θ*;
- $\left\Vert \Theta \right\Vert =\sqrt{{\sum }_{i,\,j}{\Theta }_{i,\,j}^{2}}$ is the so-called ‘Frobenius’ norm;
- *λ*_1_ and *λ*_2_ are non-negative, tunable parameters controlling the regularization strength.

The sum of the last two terms in equation 9 is a regularizer akin to the well-known Elastic Net^60^. The nuclear norm acts as an *ℓ*_1_ penalty on the singular values, promoting low rank in the solution $\widehat{\Theta }$, while the squared norm induces shrinkage in the learned coefficients like an *ℓ*_2_ penalty.

The optimization problem (equation 9) is convex (in fact, ‘strongly’ convex owing to the presence of the squared Frobenius norm). As such, it is amenable to several convex optimization solvers. For our purposes, we used the SCS solver^61^ via its Python interface available in CVXPY^62^. The SCS solver is a general-purpose conic optimization solver: it first transforms equation 9 into an equivalent conic representation (which typically yields a constrained optimization problem) and applies an operator splitting method with algorithmic embellishments, including a variant of Anderson acceleration^63^, to solve the resulting optimization problem. The SCS interface provides access to several options that control its behaviour during a solve; our code uses the default settings (see https://www.cvxgrp.org/scs/api/settings.html for a list of available settings), except for the relative and absolute feasibility tolerances, ${\epsilon }_{{\mathsf{rel}}}$ and ${\epsilon }_{{\mathsf{abs}}}$, which we set to ${\epsilon }_{{\mathsf{rel}}}={\epsilon }_{{\mathsf{abs}}}=0.005$.

### BPA extraction and community inoculation in soils

Two soils were sampled in amounts of roughly 2 kg from prairie fields (Supplementary Table 1, sites 6 and 7). Soils were sieved through to 2 mm, and a portion of each was sterilized by autoclave at 121 °C for 99 min three times, with 24 h in between each time and the last occurrence being 8 h before preparing the communities.

Of each soil, 0.85 g was suspended in 1.7 ml 100 ppm BPA medium in 48-deep-well plates and vortexed to ensure sufficient mixing of BPA. Strains were added in an identical manner for community mixing experiments. Incubation was also performed similarly. For each soil, a sample was prepared with no community added, and another sample with non-BPA medium for controls. Two technical replicates for each condition were prepared.

To measure BPA concentration at each sampling time point, BPA needed to be extracted from the soil slurry. Of the slurry, 100 μl was sampled and mixed with 150 μl 100% ethanol. The samples were then vortexed and then spun down at 2,750 *g* for 2 min. Of the supernatant, 40 μl was sampled to use for our BPA concentration assay, with an extraction efficiency of ~60% (Extended Data Fig. 8).

### Reporting summary

Further information on research design is available in the Nature Portfolio Reporting Summary linked to this article.

## Supplementary information

Supplementary Information Supplementary Discussion, Tables 1 and 2, and Figs. 1–4.

Reporting Summary

Peer Review File

## Source data

Source Data Fig. 1 Raw data for Fig. 1 (monoculture and communities time series).

Source Data Fig. 2 Raw data for Fig. 2 (raw AUC values, imputation results, and PCA components).

Source Data Fig. 3 Raw data for Fig. 3 (predicted vs measured log_10_(AUC) for all communities).

Source Data Fig. 4 Raw data for Fig. 4 (coefficient values, F1 statistics).

Source Data Fig. 5 Raw data for Fig. 5 (soil communities time series, predicted vs measured raw AUC values).

Source Data Extended Data Fig. 1 Raw data for Extended Data Fig. 1 (line of best fit for colorimetric assay, fractional errors).

Source Data Extended Data Fig. 2 Raw data for Extended Data Fig. 2 (timeseries data for random communities).

Source Data Extended Data Table1 Raw data for Extended Data Table 1 (strain taxonomy).

Source Data Extended Data Fig.3 Raw data for Extended Data Fig. 3 (CO_2_ production from aerobic respiration of BPA).

Source Data Extended Data Fig.4 Raw data for Extended Data Fig. 4 (AUCs vs community size, all communities AUCs in all concentrations, imputation optimization).

Source Data Extended Data Fig.5 Raw data for Extended Data Fig. 5 (Pearson’s correlation values, predicted vs measured log_10_(AUC) at 125 ppm).

Source Data Extended Data Fig.6 Raw data for Extended Data Fig. 6 (raw coefficients, PCA on coefficients).

Source Data Extended Data Fig.7 Raw data for Extended Data Fig. 7 (abundances of degraders and non-degraders, community size).

Source Data Extended Data Fig.8 Raw data for Extended Data Fig. 8 (soil BPA extraction efficiency).

## Figures and Tables

**Extended Data Table 1:**
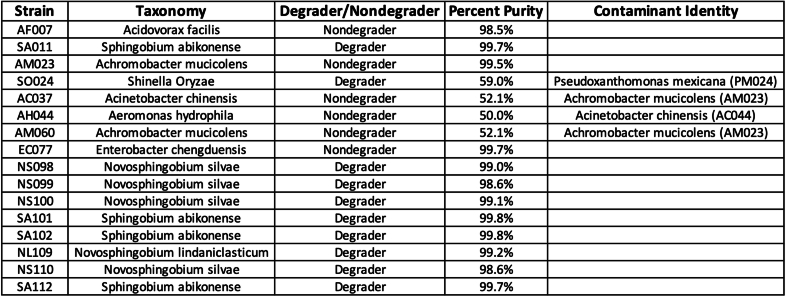
Identificaiton of All 16 Strains

Taxonomy ID and fractional purity of all sixteen strains. Percent purity is defined as the sequencing coverage representing the identified strain compared to the entire genome coverage, as reported by Plasmidsaurus. Contaminants are labeled if present.

Source data

## Data Availability

Whole genome sequencing data from our isolates, along with all raw sequence reads from the 12 synthetic consortia, are deposited under NCBI BioProject ID PRJNA1392058. All datasets generated in our study are available in the Open Science Framework (OSF) at https://osf.io/gcfr8/ (ref. ^64^). Some datasets are available in the Supplementary Tables. Source data are provided with this paper.

## Code availability

All code written for models and analysis was written in either Python or R, and is available in OSF at https://osf.io/gcfr8/ (ref. ^64^).
