# Supplementary material for: Epistatic interactions inform rational design of synthetic microbial communities for bioremediation
Source: Nat Microbiol. 2026 Jun 29;11(7):1995–2007. doi: 10.1038/s41564-026-02386-4 (PMC13323053; doi:10.1038/s41564-026-02386-4)
Supplement: Supplementary file 1 — Supplementary Discussion, Tables 1 and 2, and Figs. 1–4. [file 41564_2026_2386_MOESM1_ESM.pdf]

nature microbiology

Supplementary information https://doi.org/10.1038/s41564-026-02386-4

Epistatic interactions inform rational design of synthetic microbial communities for bioremediation

In the format provided by the authors and unedited

Supplementary Information

1 Supplementary Discussion

Regressions on Random Shuffles of log10(AUC)s

To evaluate the significance of our model fits (Fig. 3) and all resulting analyses, we sought to employ a null model of random AUCs for each community. To do this, we took each model independently and randomly shuffled the response variables 100 times. This randomization enforces no relationship between a community composition and associated AUC. We subsequently fit the model to the shuffled response variables, and ρ (Pearson's) between predicted and "true" AUC were compared to that of the original models (see Methods). The data (Fig. S1B) shows that models fit to random shuffles of the response variable perform significantly worse than the original models, where p-value < 0.01 for the lower three concentrations and p-value < 0.05 for the higher two concentrations. The result suggests that our linear models are utilizing community composition to predict AUC in a statistically significant fashion.

Increasing model order

Skwara *et al.*[11] observed that for low-ruggedness landscapes, first-order models explained most of the variance in the functions measured, while second-order coefficients improved model fits and third-order coefficients mostly did not improve model performance. However, the landscapes measured here are more rugged than those encountered by Skwara *et al.* therefore we wanted to test whether the same observations hold for this landscape.

We fit AUC data from all five initial BPA concentrations into two additional models of the form Equation 1: one only including first-order terms, and one including third-order coefficients alongside first- and second-order terms. The model including only first-order

terms was:

$$y_c^{(k)} = \beta_0^{(k)} + \sum_i \beta_i^{(k)} X_i \tag{S1}$$

while the model including third-order terms was:

$$y_c^{(k)} = \beta_0^{(k)} + \sum_i \beta_i^{(k)} X_i + \sum_{i,j} \gamma_{ij}^{(k)} X_i X_j + \sum_{i,j,k} \zeta_{ijk} X_i X_j X_k \tag{S2}$$

All models were fit in the same way and regularized with the L_2 penalty (see Methods), and model fits were compared by comparing the resulting RMSEs of leave-one-out cross-validation.

Results (Fig. S1 C) show us that, on average, adding third-order terms (green) does not substantially improve model fits compared to a model up to second-order coefficients (orange), with the only major difference seen at 125 ppm. However, we do see that fitting the data to only first-order coefficients (blue) does not lose significant predictive power compared to the second-order model, with the models fit to 100 ppm and 150 ppm data being nearly equivalent. The only major difference is seen at 60 ppm, the least epistatic model of the five, in which second-order coefficients greatly improve model performance.

Naively, it may appear that the results of Fig. S1C contradict the notion that epistasis increases with increasing BPA concentration. However, we note that the quality of the fit (RMSE, y-axis Fig. S1C) does not indicate the *relative importance or magnitude* of additive vs epistatic coefficients in the regression [64]. This is quantified via the F_1 statistic (main text).

F_1 Statistic is Qualitatively Similar with Different Metrics for BPA Degradation

One of the primary results is the rise in epistasis as BPA concentration increases - shown by the decrease in the F_1 statistic with increasing BPA concentration (Fig. 4D). However, the nonlinear nature of the AUC metric and the log-transformation performed raises the possibility that the rising epistasis may be an artifact of the chosen response variable rather than reflecting an underlying property of the data. To address this question we performed the regressions and ruggedness measurements using proxies for slopes of degradation and relative performance at maximum cross-community variance (see Methods).

Using these two alternative response variables, we fit regression models exactly as described in Methods and evaluated performance using the F_1 score. Model performance was compared to that obtained using the AUC-based response (Figures 3A and 4D). Across all metrics, the qualitative dependence of F_1 on BPA concentration was consistent (Figure S2A): inferred epistasis increased with concentration, exhibiting a sharp transition at 125 ppm. At 150 ppm, all metrics identified a dominant contribution from NL109, and inferred epistasis increased further when this effect was excluded. Pearson correlation analyses revealed comparable predictive performance across metrics, with the notable exception of 125 ppm, where only the AUC-based model retained substantial predictive power (Figure S2B). Together, these results demonstrate that the qualitative inference of epistasis is robust to the choice of response variable, while AUC-based models provide the most stable quantitative performance.

Inferred Epistasis Increases as Model Fits are Poor

The F_1 statistic (Fig. 4D) indicates that overall epistasis increases as BPA toxicity increases. However, we also note that the regression fits worsen as the initial BPA con-

centration increased (Fig. 3A). Although BPA degradation data from monocultures and communities confirms the increased importance of a community to degrade BPA at higher concentrations, we wanted to ensure that the decrease in the F_1 statistic was not an artifact of the worsening model fits with BPA concentration.

To systematically investigate this, we generated a family of landscapes with different degrees of ruggedness (controlled by different values of F_1). To evaluate how the number of sampled communities affected the estimated F_1 statistic, we generated 60 synthetic communities and randomly sampled various subsets with sample sizes ranging from 5 to 60 communities (s', see Methods). We then used the generated landscapes to compute "true" response variables, and fit the data to regression models as described in Methods. We repeated each subsampling 100 times, and the mean of all subsamplings was reported as $\hat{F}_1^{s'}$.

By systematically varying the number of sampled communities s', we assessed how well $\hat{F}_1^{s'}$ approximates the true landscape ruggedness F_1. This allowed us to quantify the effect of sample size on the accuracy of the estimated ruggedness metric and determine whether certain landscape properties (e.g., degree of epistasis) influence this relationship.

Figure S3 A shows the results of this test. As expected, we find that when all 60 communities are used in the fitting, $\hat{F}_1^{s'=60}$ closely matches that of the underlying model. However, as the sample size s' decreases, $\hat{F}_1^{s'}$ quickly becomes inaccurate. This effect is most dramatic in landscapes that are mostly or purely additive (i.e. $F_1 \approx 1$). Therefore, we conclude that highly rugged landscapes are not very susceptible to the effects of undersampling on the F_1 statistic of the inferred model.

We then wanted to ensure that the decreased F_1 statistics of the models fit to data from 125 and 150 ppm BPA are not explained by the poor model fits. We generated a single dataset X^s of size 60 communities, and then generated a single regression function F (of

the same functional form as Equation 6 with fixed β_i, $\gamma_{i,j}$) which had an F_1 statistic of 0.168. This time, we varied σ^2, the variance of the noise in η_c, to be between $\sigma'^2 = 0.0625$ and $\sigma'^2 = 5$. We generated $R = 100$ instances of noise generated from η_c for each community c, and then calculated $y_c^{\sigma'^2,r} = F(x_{c^s})|_{r=1}^{R}$. Since the response variables were of the order —1, higher values of σ'^2 represented progressively worse model fits.

Regression models were then fit to all 60 communities in X^s and all $y_c^{\sigma'^2,r}$, and the inferred $\hat{F}_1$ statistic was compared to that of F (0.168).

The results (Fig. S3 B) show that on average, the F_1 statistic *increases* as a result of poor model fits, rather than decreases. This indicates strongly that the decreased F_1 statistic seen in the 125 and 150 ppm models (Fig. 4D) are not due to the difficulty in fitting the underlying landscape.

A Low-Dimensional Regression Can Approximate the BPA Degradation Landscape

Inspired by the success of `SoftImpute` as well as the low-dimensional structure of community AUCs (Fig. 2 D), we sought to pursue a regression model that would exploit the low-dimensionality of our data to inform the regressions.

The low-rank regressor is a model that attempts to learn coefficients from all initial BPA concentrations simultaneously in a low-dimensional manner.

Model selection

We use cross-validation across BPA concentrations to select the regularization coefficients λ_1 and λ_2 (see Methods, Equation 9). Each regularization coefficient was scanned across 20 values logarithmically spaced between 0.001 and 1000. In particular, for each candidate (λ_1, λ_2) pair, we iteratively select one row of Z to act as the "held-out" community, use the remaining rows to fit $\widehat{\Theta}$ by solving Equation 9 with the given (λ_1, λ_2) pair, and compute the

RMSE score of the fit on the held-out community; finally, we compute the average RMSE scores over held-out communities (Fig. S4 bottom right panel). We select the (λ_1, λ_2) pair that achieved the lowest average score and retrain the model using all available communities. The optimal regularization coefficients were $(\lambda_1 = 4.833, \lambda_2 = 784.760)$, and the resulting $\widehat{\Theta}$ was rank-2.

Fig. S4 shows the fit of the LRR to the five BPA concentrations. In comparison to our standard linear regressions (Fig. 3), within each individual concentration, the LRR slightly underperforms the linear regression. However, the LRR has two constraints not shared with the linear regression: 1) the LRR only fits complete matrices Z and X and therefore leave-one-out cross-validation can only be performed by dropping a community out of all five concentrations simultaneously versus one-by-one, 2) the low-rank nature of $\widehat{\Theta}$ restricts the potential coefficients for all five concentrations, whereas the linear regression is free to have different parameters for each of the five concentrations. Given these restrictions, the fit of the LRR to the data is surprisingly well.

2 Supplementary Tables

Soil Sample	Latitude	Longitude	Date Sampled	Sampling Details
Soil 1	41.761921	-87.838646	10/5/22	Soil from bank of running wastewater from plastic manufacturing plant before feeding into river
Soil 2	41.82671	-87.657069	10/17/22	Soil from recycling center, on the rivershore downstream of plastic manufacturing plant
Soil 3	41.8434044	-87.6636085	10/17/22	Liquid and slurry sample from shore of river downstream from manufacturing plant and recycling center
Soil 4	41.65538739	-87.73754314	10/17/22	Soil from forest upstream of manufacturing plant and recycling center
Soil 5	41.7620076	-87.8348817	10/17/22	Soil further up the bank of running wastewater from plastic manufacturing plant before dumping into river
Soil 6	41.97780502	-87.74251079	8/26/24	Forest soil sample
Soil 7	41.57189832	-87.86993291	8/27/24	Grassland soil sample

Table S1: Data Regarding Strain Identification

Table S2: M9 media components

Reagent	Amount
5X M9 Salts (BD Biosciences, product number 248510)	5.64 g
1M MgSO4 (Fischer Scientific, product number AA3333736)	1ml
1M CaCl2 (Fischer Scientific, product number 0556)	50 μl
MiliQ Water	Remaining volume up to 500 ml
BPA (Sigma-Aldrich, product number 133072)	As needed

3 Supplementary Figures

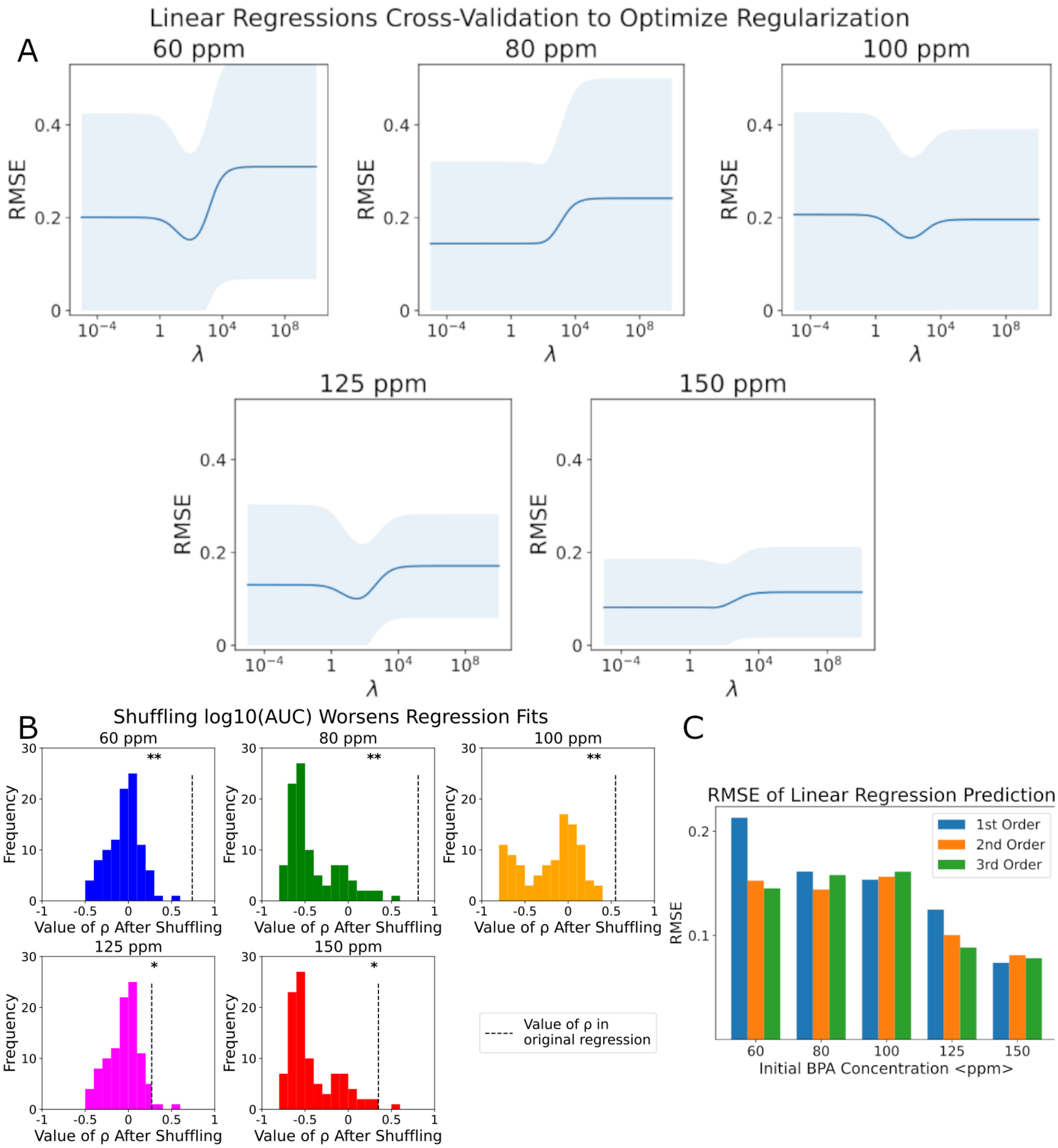

Figure S1: **Linear regression models.** See following page for caption.

Figure S1: (Continued from previous page) **(A)** Root Mean Squared Error (RMSE) (Equation 9) vs λ during leave-one-out cross-validation for all five regressions (see Methods Tbl. 1, and Fig. 3). The blue line is the average across all folds, and the shaded region is the standard deviation across all folds. The optimal penalty term was selected for each model to minimize the RMSE. **(B)** Shuffling the response variable reduces the predictive power of the model. Each panel corresponds to different initial BPA concentrations. For each concentration, we shuffled the response variable (log10(AUC)) 100 times, for each shuffle we performed a regularized linear regression as described in the Methods. The histogram shows the distribution of Pearson correlation (ρ) between measured and predicted log10(AUC) for the 100 randomizations. Dotted black lines indicate the ρ of the original regression fit (Fig. 3 A). Model fits to randomized data are significantly worse than the true data fits in Fig. 3. *p-value $< .05$ (125 ppm: p-value $= 0.02$; 150 ppm: p-value $= 0.01$;), **p-value < 0.01, calculated directly by identifying the frequency of ρ values greater than or equal to that of the original regression fit reject the null hypothesis that these randomizations perform just as well. **(C)** Root Mean Squared Error (RMSE) for each of the five concentrations fit to linear regressions of models truncated at first-order terms (β_i), second-order terms ($\gamma_{i,j}$), and third-order terms ($\zeta_{i,j,k}$). For each model at each order, leave-one-out cross-validation was done to pick the optimal penalty term to minimize out-of-sample RMSE (see Methods).

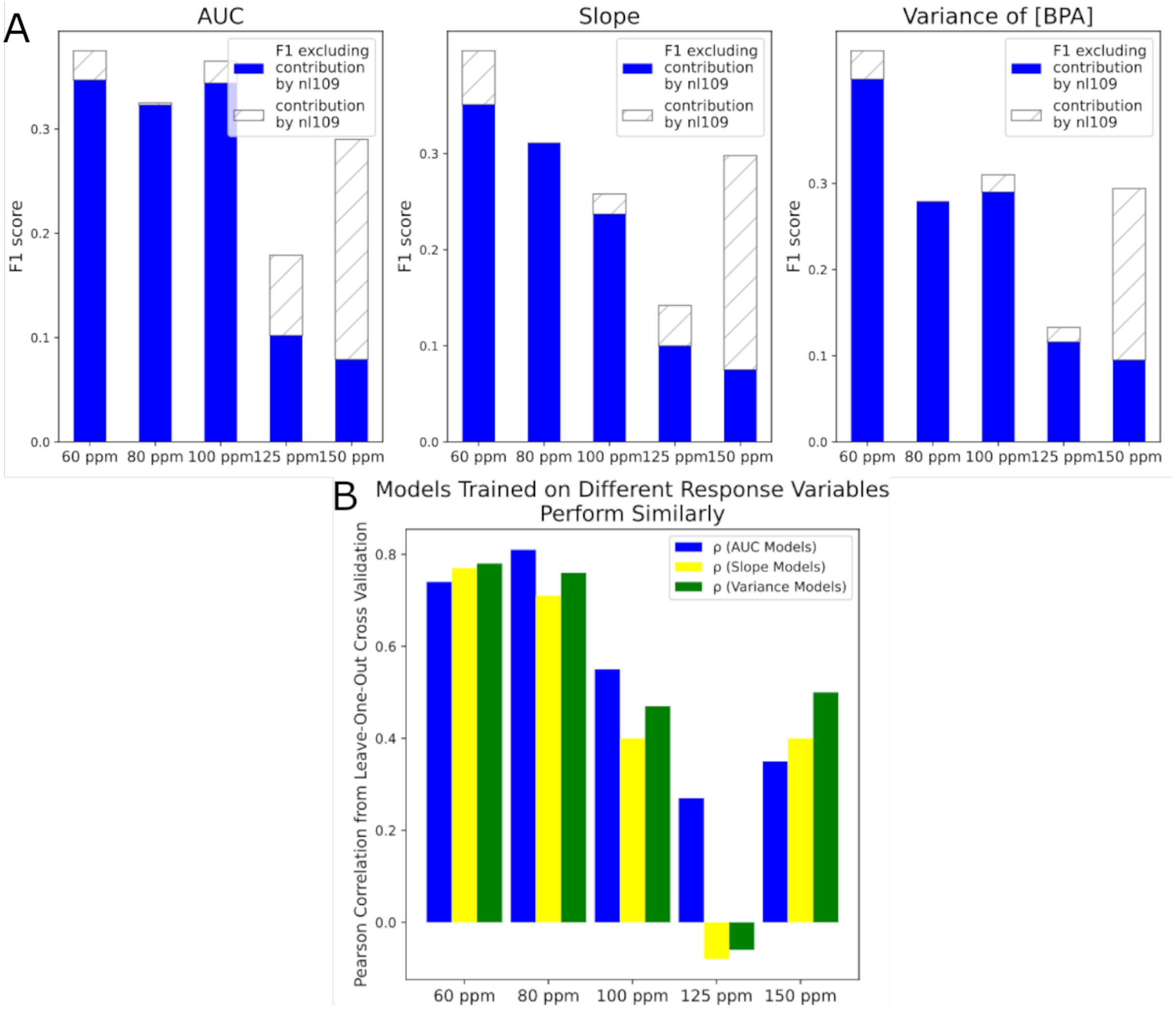

Figure S2: **Epistasis analysis is qualitatively consistent with different metrics (A)** The calculated F_1 metric for regression models computed for all five concentrations from AUC Models (left, see Fig. 4D), slope models (middle), and varaince models (right). Contribution by strain NL109 is isolated and marked in striped bars. Total F_1 is the sum of the blue and striped bars. **(B)** Comparison of Pearson's correlation for regression models fit to all five concentrations for AUC models (blue), slope models (yellow) and variance models (green).

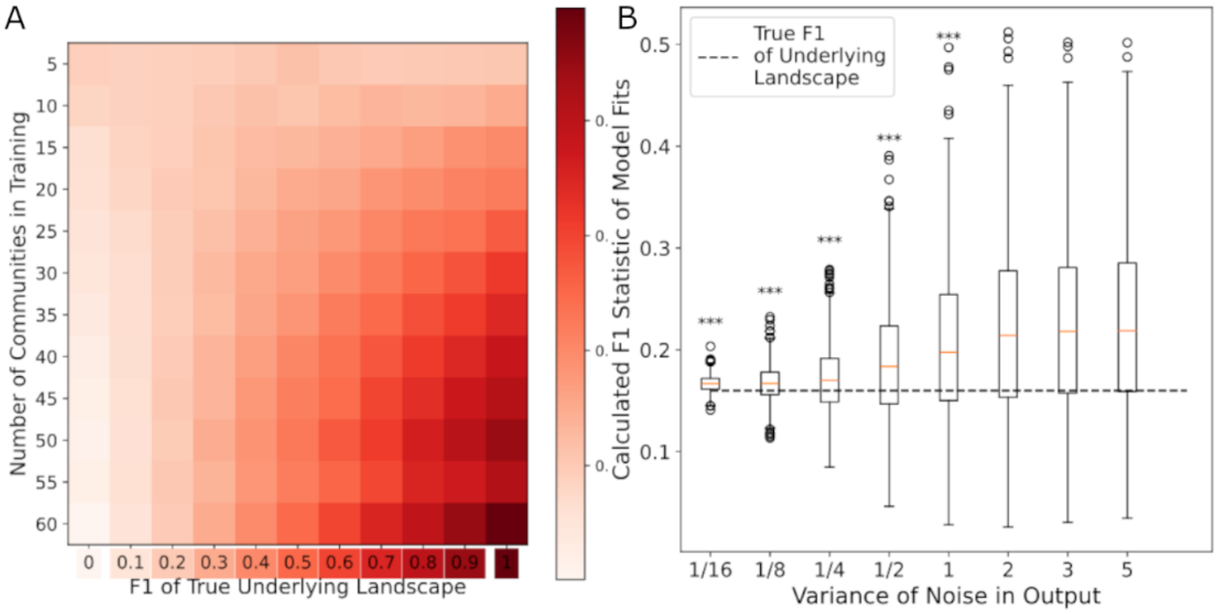

Figure S3: **Calculated F_1 statistic increases when model fits are poor (A)** Heatmap showing the calculated F_1 statistic of regression models inferred from training sets of various sizes s' and different imposed F_1 values (ruggedness), averaged across 100 random samplings. Darker colors indicate higher F_1 statistics (more additive and less epistatic). The resulting heatmap shows that F_1 can be accurately inferred as long as there are enough data points (s') but quickly becomes inaccurate when there is too little data. For all entries, the error in the inferred F_1 is less than the mean. **(B)** Effect of poor model fits (represented by increasing σ^2 of the error term η_c) on the calculated F_1 statistics. Results from 1000 iterations show that for epistatic landscapes, increasing noise also increases the inferred F_1 statistic rather than decreases it. $***p < 0.001$ as determined by a one-sided t-test rejects the null hypothesis that the mean F_1 score is greater than or equal to that of $\sigma^2 = 5$ ($\sigma^2 = \frac{1}{16}$, p-value $= 1.07 \times 10^{-90}$; $\sigma^2 = \frac{1}{8}$, p-value $= 7.15 \times 10^{-86}$; $\sigma^2 = \frac{1}{4}$, p-value $= 3.18 \times 10^{-68}$; $\sigma^2 = \frac{1}{2}$, p-value $= 1.64 \times 10^{-30}$; $\sigma^2 = 1$, p-value $= 2.34 \times 10^{-7}$).

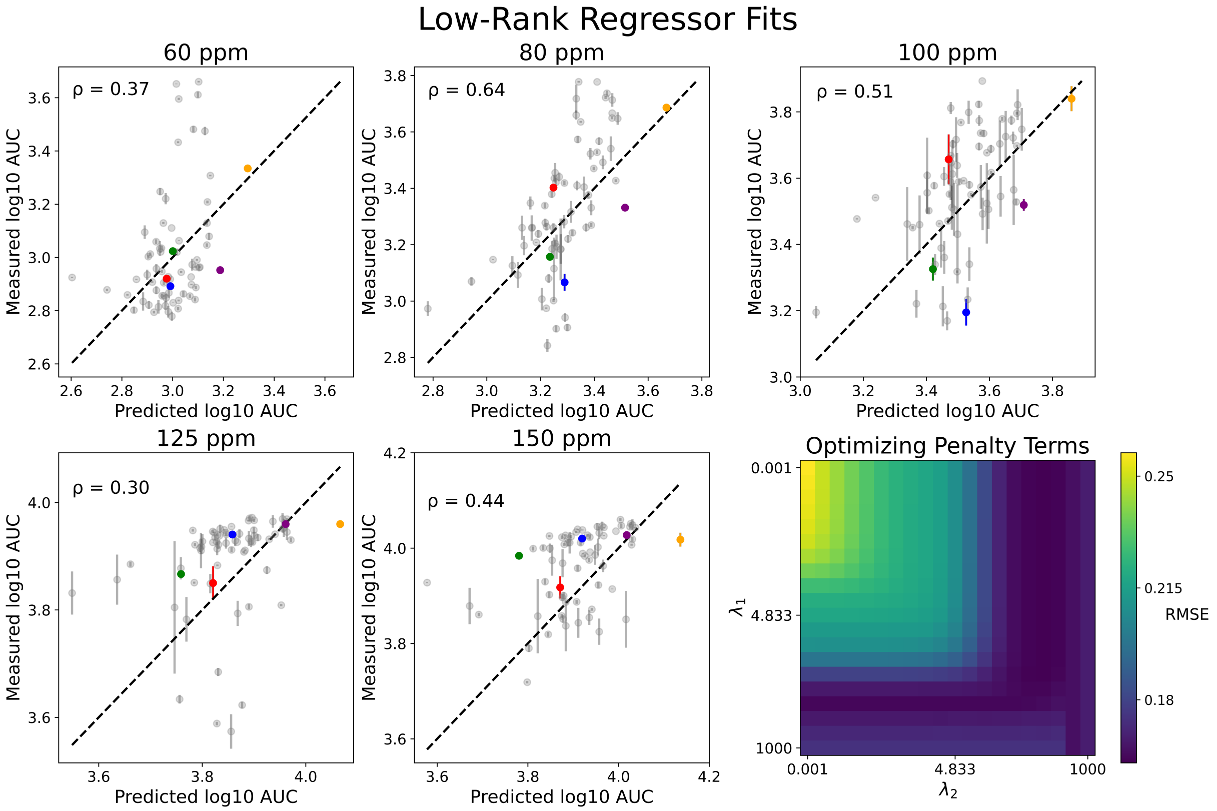

Figure S4: **Low-Rank Regressor fits to BPA AUC data** Low Rank Regressor fits to our AUC matrix (see Supplementary Information). *Top Row and Bottom Left and Center Panels* Fits for each of the five initial concentrations. Dotted black lines denote $y = x$ (perfect predictions) within each concentration. Vertical error bars represent the standard deviation of AUCs in technical replicates. *Bottom Right Panel* Total RMSE of all fits as a function of the regularization parameters λ_1 and λ_2 (Equation 9). Darker shades represent smaller RMSEs.
