# Supplementary material for: Epistatic interactions inform rational design of synthetic microbial communities for bioremediation
Source: Nat Microbiol. 2026 Jun 29;11(7):1995–2007. doi: 10.1038/s41564-026-02386-4 (PMC13323053; doi:10.1038/s41564-026-02386-4)
Supplement: Supplementary file 2 — Reporting Summary [file 41564_2026_2386_MOESM2_ESM.pdf]

nature portfolio

Corresponding author(s): Kuehn, Seppe

Last updated by author(s): 2026.04.19

Reporting Summary

Nature Portfolio wishes to improve the reproducibility of the work that we publish. This form provides structure for consistency and transparency in reporting. For further information on Nature Portfolio policies, see our Editorial Policies and the Editorial Policy Checklist.

Statistics

For all statistical analyses, confirm that the following items are present in the figure legend, table legend, main text, or Methods section.

n/a	Confirmed	
☐	☒	The exact sample size (*n*) for each experimental group/condition, given as a discrete number and unit of measurement
☐	☒	A statement on whether measurements were taken from distinct samples or whether the same sample was measured repeatedly
☐	☒	The statistical test(s) used AND whether they are one- or two-sided<br>*Only common tests should be described solely by name; describe more complex techniques in the Methods section.*
☒	☐	A description of all covariates tested
☒	☐	A description of any assumptions or corrections, such as tests of normality and adjustment for multiple comparisons
☐	☒	A full description of the statistical parameters including central tendency (e.g. means) or other basic estimates (e.g. regression coefficient) AND variation (e.g. standard deviation) or associated estimates of uncertainty (e.g. confidence intervals)
☐	☒	For null hypothesis testing, the test statistic (e.g. *F*, *t*, *r*) with confidence intervals, effect sizes, degrees of freedom and *P* value noted<br>*Give P values as exact values whenever suitable.*
☒	☐	For Bayesian analysis, information on the choice of priors and Markov chain Monte Carlo settings
☒	☐	For hierarchical and complex designs, identification of the appropriate level for tests and full reporting of outcomes
☐	☒	Estimates of effect sizes (e.g. Cohen's *d*, Pearson's *r*), indicating how they were calculated

Our web collection on statistics for biologists contains articles on many of the points above.

Software and code

Policy information about availability of computer code

Data collection	No software was used to collect data. All data was measured in-lab.
Data analysis	The python sklearn package and the R softimpute and dada2 packagse were used in our analysis. Custom code was written to fit the models and analyze the data, and a custom package was created to fit the Low Rank Regressor. Allcustom code written is available at https://osf.io/gcfr8/

For manuscripts utilizing custom algorithms or software that are central to the research but not yet described in published literature, software must be made available to editors and reviewers. We strongly encourage code deposition in a community repository (e.g. GitHub). See the Nature Portfolio guidelines for submitting code & software for further information.

Data

Policy information about availability of data

All manuscripts must include a data availability statement. This statement should provide the following information, where applicable:

- Accession codes, unique identifiers, or web links for publicly available datasets
- A description of any restrictions on data availability
- For clinical datasets or third party data, please ensure that the statement adheres to our policy

Whole genome sequencing data from our isolates, along with all raw sequence reads from the twelve synthetic consortia, are deposited under NCBI BioProject ID PRJNA1392058}. All datasets generated in our study are available within the following Open Science Framework (OSF) project: https://osf.io/gcfr8/.

Research involving human participants, their data, or biological material

Policy information about studies with human participants or human data. See also policy information about sex, gender (identity/presentation), and sexual orientation and race, ethnicity and racism.

Reporting on sex and gender	n/a
Reporting on race, ethnicity, or other socially relevant groupings	n/a
Population characteristics	n/a
Recruitment	n/a
Ethics oversight	n/a

Note that full information on the approval of the study protocol must also be provided in the manuscript.

Field-specific reporting

Please select the one below that is the best fit for your research. If you are not sure, read the appropriate sections before making your selection.

☐ Life sciences ☐ Behavioural & social sciences ☒ Ecological, evolutionary & environmental sciences

For a reference copy of the document with all sections, see nature.com/documents/nr-reporting-summary-flat.pdf

Ecological, evolutionary & environmental sciences study design

All studies must disclose on these points even when the disclosure is negative.

Study description	Our study takes 16 strains isolated from various soil samples in the Midwest, and creates 70 communities out of them to measure BPA degradation. A community function landscape is fit to the data to 1) understand what enables BPA degradation; 2) generate furhter communities.
Research sample	Our study consists of two primary research samples: 1) seven soil samples were gathered from various locations in the Midwest. Locations and descriptions of each are found in Table S2. 2) 16 bacterial isolates were obtained from enrichments of the first five soil samples for BPA degradation. These 16 were characterized and included in our community designs.
Sampling strategy	No sample size calculations were performed for obtaining our samples. Our goal was to obtain a diverse collection of isolates, including BPA degraders and those that co-exist with BPA degraders. For this, we chose a number of soil locations from areas that are potentially highly contaminated with BPA, and enriched until we obtained a diverse set of isolates that were consistent with their growth in pre-culture and their BPA degradation. Additional soil samples were obtained to test for BPA bioremediation in new environments
Data collection	Soil samples were collected from the top 4 inches of soil from all locations, with the exception of sample #3 which was a 1:1 mixture of river water and the top 2 inches of riverbank soil. All samples were collected asceptically to prevent contamination by external microbes. Bacterial isolates were collected by streaking enriched samples on solid media after sufficient BPA degradation, picking isolated colonies, and then streaking colonies to purity.
Timing and spatial scale	Timing and spatial scales are not applicable for our study design
Data exclusions	Approximately 35 total strains were isolated from our enrichments. We exlcuded strains that did not consistently grow in preculture conditions, as well as strains that did not exhibit consistent BPA degradation when inoculted in 30 ppm BPA. This lead to our final pool of 16 strains
Reproducibility	All communities were prepared in technical duplicates to ensure reproducibility in BPA degradation.
Randomization	Randomization is not applicable to our study design
Blinding	Blinding is not applicable to our study design

Did the study involve field work? ☒ Yes ☐ No

Field work, collection and transport

Field conditions	All samples were collected from natural areas in and near the Greater Chicago Area
Location	Precise locations and dates of sampling are detailed in Table S2
Access & import/export	All sampling was performed on public land, in compliance with local laws.
Disturbance	2 kg of sample was collected from all sites. The relatively small amount minimizes disturbance

Reporting for specific materials, systems and methods

We require information from authors about some types of materials, experimental systems and methods used in many studies. Here, indicate whether each material, system or method listed is relevant to your study. If you are not sure if a list item applies to your research, read the appropriate section before selecting a response.

Materials & experimental systems

n/a	Involved in the study
☒	☐ Antibodies
☒	☐ Eukaryotic cell lines
☒	☐ Palaeontology and archaeology
☐	☒ Animals and other organisms
☒	☐ Clinical data
☒	☐ Dual use research of concern
☒	☐ Plants

Methods

n/a	Involved in the study
☒	☐ ChIP-seq
☒	☐ Flow cytometry
☒	☐ MRI-based neuroimaging

Animals and other research organisms

Policy information about studies involving animals; ARRIVE guidelines recommended for reporting animal research, and Sex and Gender in Research

Laboratory animals	No laboratory animals were used in this study
Wild animals	No wild animals were used in this study
Reporting on sex	Reporting on sex is not applicable for this study
Field-collected samples	Soil samples were collected asceptically and stored individually at 4C for two to four months before processing. Bacterial strains were isolated from enrichments of these samples. More details on soil samples and bacterial isolates are found in Tables S2 and S3
Ethics oversight	The protocols used in this study were approved by the University of Chicago Institutional Biosafety Committee

Note that full information on the approval of the study protocol must also be provided in the manuscript.

Plants

Seed stocks	n/a
Novel plant genotypes	n/a
Authentication	n/a
