# Supplementary material for: Epistatic interactions inform rational design of synthetic microbial communities for bioremediation
Source: Nat Microbiol. 2026 Jun 29;11(7):1995–2007. doi: 10.1038/s41564-026-02386-4 (PMC13323053; doi:10.1038/s41564-026-02386-4)
Supplement: Supplementary file 3 — Peer Review File [file 41564_2026_2386_MOESM3_ESM.pdf]

nature portfolio

Peer Review File

Epistatic Interactions Drive Microbial Community Bioremediation and Enable Rational Design

Corresponding Author: Professor Seppe Kuehn

Version 0:

Reviewer comments:

Reviewer #1

(Remarks to the Author)
In this manuscript, the authors designed bacterial communities for BPA degradation and investigated how degradation efficiency and functional landscapes can be deduced statistically from simple growth experiments at varying BPA concentrations. Working with novel isolates from the environment, they deduce that epistasis increases with increasing toxicity, meaning that collective effects become more important. However, while the overall approach is compelling, the findings hinge on the choice of analysis for the degradation (AUC) and could significantly benefit from some low-hanging fruits to assess the validity of the predictions.
As I am no statistician, I do not comment on the appropriateness of the data or methods for the performed statistical analysis.
1. All statistical modeling is based on the area under the curve calculated from the degradation experiments. While numbers are shown in the Figures and AUC values can be downloaded, no information is given on how the AUC is calculated. Based on the figures and provided code, it seems that the authors did not normalize the AUC (e.g., as a fraction of the AUC where no degradation has occurred at all). If that is the case, it is not surprising that a higher initial BPA concentration generally results in a higher AUC since there is a dramatic baseline shift. In its current form, it is impossible to understand the sensitivity of the predictions to the choice of predictor variable.
2. Some communities comprise far more species than others (but have an equal individual inoculum of OD 0.02), which changes the total inoculum size (e.g., mix8 must have fewer than 3 times as many bacteria compared to mix20 initially). Have the authors examined the impact of inoculum size on degradation rate/lag-phase?
3. Understanding the individual contribution or growth of the species for each community is informative to benchmarking the model's performance. Assessing the absolute or relative abundance of the strains after the experiment and comparing them to the average effect of individual strains (beta) may provide a critical comparison to assess the model's effectiveness (if degradation translates to biomass increase). Currently, there is no way to gauge the performance of the model effectively.
4. The authors included non-degraders in many of the communities, which I found intriguing. More information on their importance for degradation, and e.g., their final abundance, would be appreciated. I can see that the authors highlighted the non-degrader-non-degrader coefficients on page 16, but a deeper dive into their possible importance by looking at the final community composition (do they still show up?) would be helpful.

Minor comments:
1. In what way does the soft impute outperform simple interpolation for the different communities? If I understood correctly, soft impute predicts the degradation of an individual community at a specific concentration based on the observed degradation at other concentrations. How does that compare to simple regression or interpolation?
2. What is the reason for using log(AUC) instead of AUC as a predictor for the functional landscapes?
3. Figure 2D: Despite similar degradation trends in the lower concentrations (e.g., 60 ppm and 80 ppm), there is a stark difference in the AUC. How would these look like with normalized AUC?
4. The concept of epistasis in terms of community interaction needs a clearer introduction. Interpretation and translation to “community relevance” is difficult otherwise.
5. Figure 4D: This information may be valuable earlier in the manuscript.
6. Figure 5: Do the authors know why po109 seems to be important? Is it present in the final community? Also, since F1 is strongly increasing again from 125 ppm to 150 ppm, does the trend of increasing epistatic effects hold?

(Remarks on code availability)

Reviewer #2

(Remarks to the Author)
This manuscript presents a well-structured study demonstrating the application of community-function landscape modeling to guide the rational design of synthetic microbial consortia. Using bisphenol A (BPA) degradation as a case study, the authors isolate a library of soil bacteria, quantify their degradation performance in multiple combinations and under varying contaminant concentrations, and construct statistical models that capture both additive and epistatic contributions to community function. They validate these predictions both in vitro and in soil microcosms, showing that the designed communities can outperform the native microbiota in degrading BPA. The study is conceptually innovative, methodologically sound, and offers a compelling proof of principle for predictive microbiome engineering.

The strengths of the work are the following. The experimental design is rigorous, with careful control over community composition and contaminant conditions. The modeling approach is clearly described and implemented, effectively capturing key features of microbial interaction networks that shape biodegradation outcomes. The ability to transition from in vitro predictions to performance validation in soil, a complex and more ecologically realistic environment, enhances the practical relevance of the findings. Overall, the manuscript is clearly written and presents a coherent narrative from hypothesis to validation.

Nonetheless, there are a limitations that merit deeper consideration in the discussion. First, while the soil experiments are a valuable step toward real-world application, natural environments are typically far more complex. Autochthonous microbial communities in diverse soils or aquatic systems may compete with, inhibit, or even displace introduced consortia. A more detailed discussion on how the inoculated synthetic community might be influenced by native microbiota and environmental variability would strengthen the translational perspective of the study.

Second, the exclusive focus on BPA as a single target contaminant limits the generalizability of the approach. In practice, polluted environments often contain mixtures of compounds—such as petroleum fractions may interact synergistically or antagonistically, complicating biodegradation. While it is reasonable to begin with a defined substrate, the authors should acknowledge that extending their method to multiple contaminants will pose additional challenges in both experimental design and modeling complexity.

Finally, the study does not report the composition of the synthetic consortia after the biodegradation experiments. Without data on the final community structure—whether through sequencing, plating, or another method—it is difficult to assess whether all introduced strains remained active, or if certain taxa dominated the degradation process. If such analysis is not feasible at this stage, the authors should at least discuss this as a limitation.

(Remarks on code availability)

Decision Letter:

23rd May 2025

Dear Seppe,

Thank you for your patience while your manuscript "Epistatic Interactions Drive Microbial Community Bioremediation and Enable Rational Design" was under peer review at Nature Microbiology. I'm very sorry that I've got the unfortunate job of being the bearer of bad news about this submission. It has now been seen by our referees, whose expertise and comments you will find at the end of this email, and in light of their advice, we have decided that we cannot offer to publish your manuscript in Nature Microbiology.

From the reports, you will see that the referees do agree that this approach to bioremediation is a laudable idea. However, they also raise serious concerns about methodology and the level of validation, as well as with the general applicability of the approach (because of only BPA was tested, and because there were not more "environmentally relevant" conditions tested). After discussing these criticisms with the team, we felt that these criticisms are sufficiently important as to preclude publication of your work in Nature Microbiology, as they highlight that the work remains too preliminary at this stage.

I have discussed your manuscript and the reviewers’ comments with our colleagues at Nature Communications. They would send the appropriately revised version to the original reviewers (if they are available). Should you wish to have your revised paper considered by Nature Communications, please use the link to the Springer Nature manuscript transfer service in the footnote once the revision is ready, and include a point-by-point response to the reviewers’ concerns.

Please note that Nature Communications are satisfied that the findings represent a sufficient advance for publication. However, they will require all technical concerns, in particular about insufficient validation, unclear sensitivity of predictions and insufficient analysis of the final community composition raised by the referees to be addressed in full before sending the revised paper back to the original referees. Please note that the editors would likely also involve an additional reviewer.

Your handling editor at Nature Communications would be Dr. Danielle Troppens (danielle.troppens@nature.com). If there is anything you would like to discuss before transferring the paper and its reviews, please don’t hesitate to contact her by e-mail.

Please note that Nature Communications is a fully open access journal. For information about article processing charges, open access funding, and advice and support from Springer Nature, please consult the Nature Communications Open Access page (www.nature.com/ncomms/open_access/index.html).

To transfer your manuscript please use our manuscript transfer portal. You will not have to re-supply manuscript metadata and files, unless you wish to make modifications. For more information, please see our manuscript transfer FAQ page.

Once again, I am sorry that we cannot be more positive on this occasion, but hope that you find the referees' comments helpful when preparing your paper for resubmission elsewhere.

Yours sincerely,

****
Reviewer Expertise:

Referee #1: environmental microbiology, biogeochemistry, ecological and metabolic networks
Referee #2: environmental microbiology, bioremediation

Reviewers Comments:

Reviewer #1 (Remarks to the Author):

In this manuscript, the authors designed bacterial communities for BPA degradation and investigated how degradation efficiency and functional landscapes can be deduced statistically from simple growth experiments at varying BPA concentrations. Working with novel isolates from the environment, they deduce that epistasis increases with increasing toxicity, meaning that collective effects become more important. However, while the overall approach is compelling, the findings hinge on the choice of analysis for the degradation (AUC) and could significantly benefit from some low-hanging fruits to assess the validity of the predictions. As I am no statistician, I do not comment on the appropriateness of the data or methods for the performed statistical analysis.
1. All statistical modeling is based on the area under the curve calculated from the degradation experiments. While numbers are shown in the Figures and AUC values can be downloaded, no information is given on how the AUC is calculated. Based on the figures and provided code, it seems that the authors did not normalize the AUC (e.g., as a fraction of the AUC where no degradation has occurred at all). If that is the case, it is not surprising that a higher initial BPA concentration generally results in a higher AUC since there is a dramatic baseline shift. In its current form, it is impossible to understand the sensitivity of the predictions to the choice of predictor variable.
2. Some communities comprise far more species than others (but have an equal individual inoculum of OD 0.02), which changes the total inoculum size (e.g., mix8 must have fewer than 3 times as many bacteria compared to mix20 initially). Have the authors examined the impact of inoculum size on degradation rate/lag-phase?
3. Understanding the individual contribution or growth of the species for each community is informative to benchmarking the model's performance. Assessing the absolute or relative abundance of the strains after the experiment and comparing them to the average effect of individual strains (beta) may provide a critical comparison to assess the model's effectiveness (if degradation translates to biomass increase). Currently, there is no way to gauge the performance of the model effectively.
4. The authors included non-degraders in many of the communities, which I found intriguing. More information on their importance for degradation, and e.g., their final abundance, would be appreciated. I can see that the authors highlighted the non-degrader-non-degrader coefficients on page 16, but a deeper dive into their possible importance by looking at the final community composition (do they still show up?) would be helpful.

Minor comments:
1. In what way does the soft impute outperform simple interpolation for the different communities? If I understood correctly, soft impute predicts the degradation of an individual community at a specific concentration based on the observed degradation at other concentrations. How does that compare to simple regression or interpolation?
2. What is the reason for using log(AUC) instead of AUC as a predictor for the functional landscapes?
3. Figure 2D: Despite similar degradation trends in the lower concentrations (e.g., 60 ppm and 80 ppm), there is a stark difference in the AUC. How would these look like with normalized AUC?
4. The concept of epistasis in terms of community interaction needs a clearer introduction. Interpretation and translation to “community relevance” is difficult otherwise.
5. Figure 4D: This information may be valuable earlier in the manuscript.
6. Figure 5: Do the authors know why po109 seems to be important? Is it present in the final community? Also, since F1 is strongly increasing again from 125 ppm to 150 ppm, does the trend of increasing epistatic effects hold?

Reviewer #2 (Remarks to the Author):

This manuscript presents a well-structured study demonstrating the application of community-function landscape modeling to guide the rational design of synthetic microbial consortia. Using bisphenol A (BPA) degradation as a case study, the authors isolate a library of soil bacteria, quantify their degradation performance in multiple combinations and under varying contaminant concentrations, and construct statistical models that capture both additive and epistatic contributions to community function.

They validate these predictions both in vitro and in soil microcosms, showing that the designed communities can outperform the native microbiota in degrading BPA. The study is conceptually innovative, methodologically sound, and offers a compelling proof of principle for predictive microbiome engineering.

The strengths of the work are the following. The experimental design is rigorous, with careful control over community composition and contaminant conditions. The modeling approach is clearly described and implemented, effectively capturing key features of microbial interaction networks that shape biodegradation outcomes. The ability to transition from in vitro predictions to performance validation in soil, a complex and more ecologically realistic environment, enhances the practical relevance of the findings. Overall, the manuscript is clearly written and presents a coherent narrative from hypothesis to validation.

Nonetheless, there are a limitations that merit deeper consideration in the discussion. First, while the soil experiments are a valuable step toward real-world application, natural environments are typically far more complex. Autochthonous microbial communities in diverse soils or aquatic systems may compete with, inhibit, or even displace introduced consortia. A more detailed discussion on how the inoculated synthetic community might be influenced by native microbiota and environmental variability would strengthen the translational perspective of the study.

Second, the exclusive focus on BPA as a single target contaminant limits the generalizability of the approach. In practice, polluted environments often contain mixtures of compounds—such as petroleum fractions may interact synergistically or antagonistically, complicating biodegradation. While it is reasonable to begin with a defined substrate, the authors should acknowledge that extending their method to multiple contaminants will pose additional challenges in both experimental design and modeling complexity.

Finally, the study does not report the composition of the synthetic consortia after the biodegradation experiments. Without data on the final community structure—whether through sequencing, plating, or another method—it is difficult to assess whether all introduced strains remained active, or if certain taxa dominated the degradation process. If such analysis is not feasible at this stage, the authors should at least discuss this as a limitation.

****

Version 1:

Decision Letter:

25th June 2025

Dear Seppe,

Thank you for your letter asking us to reconsider our decision on your Article entitled "Epistatic Interactions Drive Microbial Community Bioremediation and Enable Rational Design". After careful consideration we have decided that we would be willing to consider a fully revised version of your manuscript that addresses the concerns of the reviewers. After discussing with my colleagues, we have agreed that we would not hold you to testing this in the field, or assessing a suite of other contaminants-- though we do agree that it would be great to discuss these things in the revised manuscript.

Along with your revised manuscript, you should also submit a separate point-by-point response to all of the concerns raised by the referees, in each case describing what changes have been made to the manuscript or, alternatively, if no action has been taken, providing a compelling argument for why that is the case.

Once you resubmit, if we feel that a substantial attempt has been made to address the referees' comments, this response will be sent back to the referees - along with the revised manuscript - so that they can judge whether their concerns have been addressed satisfactorily or otherwise.

When revising your paper:

- ensure it complies with our format requirements for Letters as set out in our guide to authors at www.nature.com/nmicrobiol/authors/index.html

- state in a cover note the length of the text, methods and legends; the number of references and the number of display items.

Please ensure that all correspondence is marked with your Nature Microbiology reference number in the subject line.

Please use the following link to submit your revised manuscript:

Link Redacted

We hope to receive your revised paper within four weeks. If you cannot send it within this time, please let us know so that we can close your file. In this event, we will still be happy to reconsider your paper at a later date so long as nothing similar has been

accepted for publication at Nature Microbiology or published elsewhere in the meantime. Should you miss the four-week deadline and your paper is eventually published, the received date will be that of the revised, not the original, version.

I would appreciate it if you could tell me if you think you will be able to submit a revised manuscript, and also the likely timescale.

I look forward to hearing from you soon.

Yours sincerely,

Version 2:

Reviewer comments:

Reviewer #1

(Remarks to the Author)
I thank the editor for the opportunity to review the manuscript NMICROBIOL-25031102B by Yousef et al. for a second time and commend the authors for their efforts to improve the manuscript. Similar to the first round of reviews, I thoroughly enjoyed reading about the work and feel like the authors performed valuable follow-up experiments. While the inability to distinguish all strains via amplicon sequencing and the contamination of 4 out of 16 isolates are unfortunate, I appreciate the transparency with which the authors have approached this hurdle. My view aligns with the authors' that this contamination does not negate the main findings of the study regarding the emergence of epistasis. Ensuring that isolates are genetically tractable for facile quantification of their relative abundanced may provide great insights in future studies.

(Remarks on code availability)
As I am not a statistician, I did not review the code in detail. I accessed the repository to verify the presence of data files and sequences.

Reviewer #2

(Remarks to the Author)
The authors have adequately resolved the issues highlighted in the previous review round. I am therefore in favor of publication of the manuscript in its present version.

(Remarks on code availability)
regarding the OSF repository: although I did not execute the code, it is clearly of high quality. The authors provided a modular Python package alongside R scripts, all featuring clear installation instructions. Overall, the repository is well-documented and designed with reproducibility in mind.

Decision Letter:

Our ref: NMICROBIOL-25031102B

11th March 2026

Dear Seppe,

Thank you for submitting your revised manuscript "Epistatic Interactions Drive Microbial Community Bioremediation and Enable Rational Design" (NMICROBIOL-25031102B). It has now been seen by the original referees and their comments are below. The reviewers find that the paper has improved in revision, and therefore we'll be happy in principle to publish it in Nature Microbiology, pending minor revisions to satisfy the referees' final requests and to comply with our editorial and formatting guidelines.

If the current version of your manuscript is in a PDF format, please email us a copy of the file in an editable format (Microsoft Word or LaTex)-- we can not proceed with PDFs at this stage.

We are now performing detailed checks on your paper and will send you a checklist detailing our editorial and formatting requirements in about a week. Please do not upload the final materials and make any revisions until you receive this additional information from us.

Thank you again for your interest in Nature Microbiology Please do not hesitate to contact me if you have any questions.

Sincerely,

Reviewer #1 (Remarks to the Author):

I thank the editor for the opportunity to review the manuscript NMICROBIOL-25031102B by Yousef et al. for a second time and commend the authors for their efforts to improve the manuscript. Similar to the first round of reviews, I thoroughly enjoyed reading about the work and feel like the authors performed valuable follow-up experiments. While the inability to distinguish all strains via amplicon sequencing and the contamination of 4 out of 16 isolates are unfortunate, I appreciate the transparency with which the authors have approached this hurdle. My view aligns with the authors' that this contamination does not negate the main findings of the study regarding the emergence of epistasis. Ensuring that isolates are genetically tractable for facile quantification of their relative abundanced may provide great insights in future studies.

Reviewer #1 (Remarks on code availability):

As I am not a statistician, I did not review the code in detail. I accessed the repository to verify the presence of data files and sequences.

Reviewer #2 (Remarks to the Author):

The authors have adequately resolved the issues highlighted in the previous review round. I am therefore in favor of publication of the manuscript in its present version.

Reviewer #2 (Remarks on code availability):

regarding the OSF repository: although I did not execute the code, it is clearly of high quality. The authors provided a modular Python package alongside R scripts, all featuring clear installation instructions. Overall, the repository is well-documented and designed with reproducibility in mind.

Version 3:

Decision Letter:

12th May 2026

Dear Seppe,

I hope you're doing well! Some good news:

I am pleased to accept your Article "Epistatic Interactions Drive Microbial Community Bioremediation and Enable Rational Design" for publication in Nature Microbiology. Thank you for having chosen to submit your work to us and many congratulations.

Over the next few weeks, your paper will be copyedited to ensure that it conforms to Nature Microbiology style. We look particularly carefully at the titles of all papers to ensure that they are relatively brief and understandable.

Once your paper is typeset, you will receive an email with a link to choose the appropriate publishing options for your paper and our Author Services team will be in touch regarding any additional information that may be required. Once your paper has been scheduled for online publication, the Nature press office will be in touch to confirm the details.

You may wish to make your media relations office aware of your accepted publication, in case they consider it appropriate to organize some internal or external publicity. Once your paper has been scheduled you will receive an email confirming the publication details. This is normally 3-4 working days in advance of publication. If you need additional notice of the date and time of publication, please let the production team know when you receive the proof of your article to ensure there is sufficient time to coordinate. Further information on our embargo policies can be found here:
https://www.nature.com/authors/policies/embargo.html

After the grant of rights is completed, you will receive a link to your electronic proof via email with a request to make any corrections within 48 hours. If, when you receive your proof, you cannot meet this deadline, please inform us at rjsproduction@springernature.com immediately. You will not receive your proofs until the publishing agreement has been received through our system

Due to the importance of these deadlines, we ask you please us know now whether you will be difficult to contact over the next month. If this is the case, we ask you provide us with the contact information (email, phone and fax) of someone who will be able to check the proofs on your behalf, and who will be available to address any last-minute problems.

Acceptance of your manuscript is conditional on all authors' agreement with our publication policies (see https://www.nature.com/nmicrobiol/editorial-policies). In particular your manuscript must not be published elsewhere.

Authors may need to take specific actions to achieve compliance with funder and institutional open access mandates. If

your research is supported by a funder that requires immediate open access (e.g. according to Plan S principles or the NIH public access policy) then you should select the gold OA route, and we will direct you to the compliant route where possible. Because authors warrant under our subscription licensing terms that they haven’t committed to licensing any version of their article under a licence inconsistent with the terms of our agreement – including the applicable embargo period – publication under the subscription model isn’t suitable for authors whose funders require no embargo.

If you have any questions about our publishing options, costs, Open Access requirements, or our legal forms, please contact ASJournals@springernature.com

An online order form for reprints of your paper is available at https://www.nature.com/reprints/author-reprints.html. All co-authors, authors' institutions and authors' funding agencies can order reprints using the form appropriate to their geographical region.

We welcome the submission of potential cover material (including a short caption of around 40 words) related to your manuscript; suggestions should be sent to Nature Microbiology as electronic files (the image should be 300 dpi at 210 x 297 mm in either TIFF or JPEG format). Please note that such pictures should be selected more for their aesthetic appeal than for their scientific content, and that colour images work better than black and white or grayscale images. Please do not try to design a cover with the Nature Microbiology logo etc., and please do not submit composites of images related to your work. I am sure you will understand that we cannot make any promise as to whether any of your suggestions might be selected for the cover of the journal.

You can now use a single sign-on for all your accounts, view the status of all your manuscript submissions and reviews, access usage statistics for your published articles and download a record of your refereeing activity for the Nature journals.

To assist our authors in disseminating their research to the broader community, our SharedIt initiative provides you with a unique shareable link that will allow anyone (with or without a subscription) to read the published article. Recipients of the link with a subscription will also be able to download and print the PDF.

As soon as your article is published, you will receive an automated email with your shareable link.

With kind regards,

P.S. Click on the following link if you would like to recommend Nature Microbiology to your librarian http://www.nature.com/subscriptions/recommend.html#forms

** Visit the Springer Nature Editorial and Publishing website at www.springernature.com/editorial-and-publishing-jobs for more information about our career opportunities. If you have any questions please click here.**

Open Access This Peer Review File is licensed under a Creative Commons Attribution 4.0 International License, which permits use, sharing, adaptation, distribution and reproduction in any medium or format, as long as you give appropriate credit to the original author(s) and the source, provide a link to the Creative Commons license, and indicate if changes were made.
In cases where reviewers are anonymous, credit should be given to 'Anonymous Referee' and the source.
The images or other third party material in this Peer Review File are included in the article's Creative Commons license, unless indicated otherwise in a credit line to the material. If material is not included in the article's Creative Commons license and your intended use is not permitted by statutory regulation or exceeds the permitted use, you will need to obtain permission directly from the copyright holder.
To view a copy of this license, visit https://creativecommons.org/licenses/by/4.0/

Reviewer 1

Comment #1: All statistical modeling is based on the area under the curve calculated from the degradation experiments. While numbers are shown in the Figures and AUC values can be downloaded, no information is given on how the AUC is calculated. Based on the figures and provided code, it seems that the authors did not normalize the AUC (e.g., as a fraction of the AUC where no degradation has occurred at all). If that is the case, it is not surprising that a higher initial BPA concentration generally results in a higher AUC since there is a dramatic baseline shift. In its current form, it is impossible to understand the sensitivity of the predictions to the choice of predictor variable.

Response #1: We believe this comment stems from a technical confusion which would be easily addressed through minor revision. As the reviewer points out, higher BPA will trivially lead to higher AUC. However, our SoftImpute and Regression models account for this through z-scoring (mean-centering and dividing by the standard deviation) every entry within each column, such that the response variable for each concentration is has a mean of 0 and a standard deviation of 1, as discussed in Methods. This allows the SoftImpute results to be interpretable despite the differences in baselines, and allows for a direct comparison of model coefficients between different concentrations. We will make this point clearer in our explanation of results.

Second, we have explored how different response variables (such as time to degrade to a fixed concentration, or average rate of BPA degradation) affect our conclusions, and we found that our qualitative results remain the same. A revision will include this data and analysis within the supplemental discussions.

Comment #2: Some communities comprise far more species than others (but have an equal individual inoculum of OD 0.02), which changes the total inoculum size (e.g., mix8 must have fewer than 3 times as many bacteria compared to mix20 initially). Have the authors examined the impact of inoculum size on degradation rate/lag-phase?

Response #2: Our rationale for using a fixed OD per strain grounded in our community-function landscape analysis. In our regressions, we estimate the average impact of each strain on AUC. As a result, we do not wish to vary the initial abundances of a strain in a manner that depends on the community it is added to. If we did reduce the initial density of strains in proportion to community richness (e.g. to have fixed total OD in all communities) this would cause the effect of each community member to depend on community richness. Instead, our goal is to estimate the effects of each strain, added identically to each community. As a result of this rationale, we have not systematically studied the dependence of lag phase or rate on inoculum size.

Further, it is important to recognize that richness is not the dominant contributor to community function in most conditions. In particular, Mix20 our largest community, is not the fastest degrading community in any of the five initial concentrations of BPA. This informs us of the

importance of epistasis for BPA degradation, where interactions that are both beneficial and harmful for community function exist. It also shows that richness is not the dominant factor in determining degradation rate (see below). Consistent with our findings, a significant correlation between community size and BPA degradation is found at 60 ppm, where additive effects dominate degradation; and 150 ppm, where one strain has a very large additive effect. Both of these effects are captured by our statistical model.

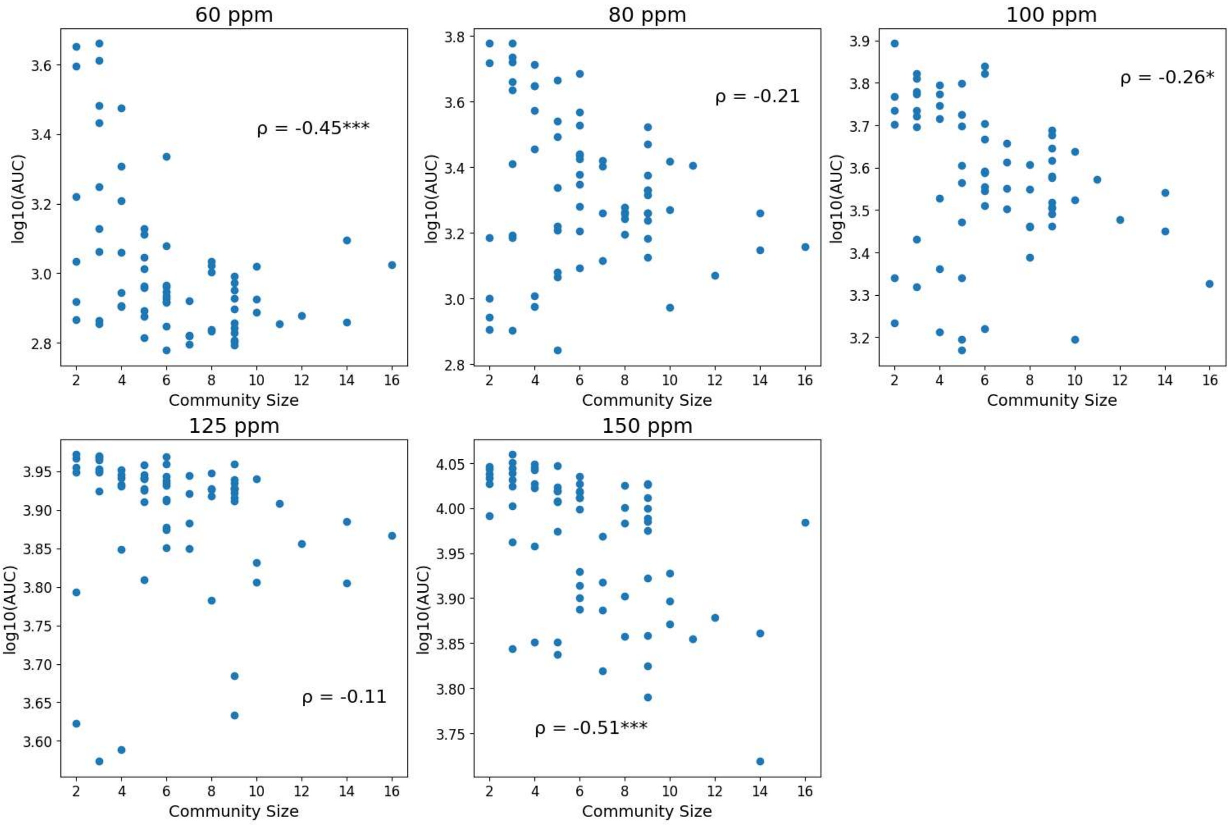

*p-value < 0.05, ***p-value < 0.001. Mix20 is the only community with a size of 16 (furthest point to the right in each plot).

Comment #3: Understanding the individual contribution or growth of the species for each community is informative to benchmarking the model's performance. Assessing the absolute or relative abundance of the strains after the experiment and comparing them to the average effect of individual strains (beta) may provide a critical comparison to assess the model's effectiveness (if degradation translates to biomass increase). Currently, there is no way to gauge the performance of the model effectively.

Response #3: We find this comment useful. **We would be willing to perform such sequencing measurements in any revision for Nature Microbiology**. We do note, however, that the model's effectiveness was already established via careful statistical validation and out of sample predictions. We also note that the relationship between the regression coefficients and abundances is far from trivial. First, the regression estimates *background average effects* of

individual strains on BPA. Optical density measurements reveal little to no change in biomass over the course of the experiment. This holds true for single strain BPA degradation, despite the measurable production of carbon dioxide as shown in the SI. Therefore, it may very well be that BPA catabolism is supporting maintenance energy for the degrading strains and those that degrade well simply die less quickly. Sequencing measurements could reveal such dynamics, but due to these considerations, we decided to not perform such experiments prior to an initial submission.

Comment #4: The authors included non-degraders in many of the communities, which I found intriguing. More information on their importance for degradation, and e.g., their final abundance, would be appreciated. I can see that the authors highlighted the non-degrader-non-degrader coefficients on page 16, but a deeper dive into their possible importance by looking at the final community composition (do they still show up?) would be helpful.

Response #4: We agree with the reviewer that relative abundance data would be critical for a deeper exploration in the role of nondegraders. As in our response to the previous comment, we will measure relative abundance of strains in our communities and analyze the abundance of nondegraders.

Minor Comment #1: In what way does the soft impute outperform simple interpolation for the different communities? If I understood correctly, soft impute predicts the degradation of an individual community at a specific concentration based on the observed degradation at other concentrations. How does that compare to simple regression or interpolation?

Response #5: Figure S8 shows that soft impute performs on par if slightly worse than a regression approach. Predicting the mean for each concentration is worse than both regressions and SoftImpute The relatively high performance for SoftImpute is not surprising given that SoftImpute is performing a simpler prediction than full regression (e.g. landscape inference), since it is given information about how each community performs at 4 other concentrations to predict performance at a target concentration. In contrast, regressions are only given community composition to perform predictions. Regressions have greater flexibility (more degrees of freedom depending on regularization strength). So we believe Figure S8 addresses this question. We are unclear on what the referee means by "interpolation" – are you suggesting a polynomial approximation to AUC across concentrations for a given community? Does this differ in a qualitative way from inferring the landscape Equation from strain presence/absence?

Minor Comment #2: What is the reason for using log(AUC) instead of AUC as a predictor for the functional landscapes?

Response #6: log(AUC) is a variance stabilizing transformation that is commonly employed in regression problems to put wide ranging response variables on a common scale. The AUCs of our data range from ~800 to ~11000. At this range, the loss function of the regression will strongly favor communities with larger AUC to keep losses low. Using log(AUC) puts all of the

THE UNIVERSITY OF CHICAGO

data within the same order of magnitude (~2.9 to ~4.1), alleviating this effect. Using log-transformed data is standard practice in this situation.

Minor Comment #3: Figure 2D: Despite similar degradation trends in the lower concentrations (e.g., 60 ppm and 80 ppm), there is a stark difference in the AUC. How would these look like with normalized AUC?

Response #7: While the AUC is different between the two concentrations, this difference is of no concern to the models, given the z-scoring that is performed on every column before performing the imputation (see our response to comment #1).

Minor Comment #4: The concept of epistasis in terms of community interaction needs a clearer introduction. Interpretation and translation to "community relevance" is difficult otherwise.

Response #8: We thank the reviewer for this comment and will address the concept of epistasis more cleanly in the introduction.

Minor Comment #5: Figure 4D: This information may be valuable earlier in the manuscript.

Response #9: We agree with the reviewer and will move this subpanel to Figure 1

Minor Comment #6: Do the authors know why po109 seems to be important? Is it present in the final community? Also, since F1 is strongly increasing again from 125 ppm to 150 ppm, does the trend of increasing epistatic effects hold?

Response #10: Currently, we do not have a mechanistic understanding of the importance of PO109 in toxic concentrations of BPA. We believe that relative abundance data that we will collect per our responses to comments 3 and 4 will be critical to revealing some of this information.

Reviewer #2

Comment #1: while the soil experiments are a valuable step toward real-world application, natural environments are typically far more complex. Autochthonous microbial communities in diverse soils or aquatic systems may compete with, inhibit, or even displace introduced consortia. A more detailed discussion on how the inoculated synthetic community might be influenced by native microbiota and environmental variability would strengthen the translational perspective of the study.

Response #1: We agree with the reviewer that natural environments are far more complex than the soil experiments we performed. The theory posited by Silverstein *et al.* on niche specialization explains the success of community inoculation in our case, but we agree that we

cannot sufficiently generalize our success to the complicated natural microbiomes that exist. We will expand on this point in the discussion.

Comment #2: the exclusive focus on BPA as a single target contaminant limits the generalizability of the approach. In practice, polluted environments often contain mixtures of compounds—such as petroleum fractions may interact synergistically or antagonistically, complicating biodegradation. While it is reasonable to begin with a defined substrate, the authors should acknowledge that extending their method to multiple contaminants will pose additional challenges in both experimental design and modeling complexity.

Response #2: We agree with the reviewer that our approach will need to be extended to account for multiple contaminants, which are often the case in reality. We will discuss this limitation in the discussion.

Comment #3: the study does not report the composition of the synthetic consortia after the biodegradation experiments. Without data on the final community structure—whether through sequencing, plating, or another method—it is difficult to assess whether all introduced strains remained active, or if certain taxa dominated the degradation process. If such analysis is not feasible at this stage, the authors should at least discuss this as a limitation

Response #3: We thank the reviewer for this comment. A key advantage to our approach is that the initial community composition is sufficient to explain the community function. However, we agree that information on changes in community structure at the endpoint of degradation provides key information on important contributions to the degradation process. In concordance with our responses to reviewer #1, we will analyze endpoint relative abundance data and observe whether changes in relative abundance correlate with model coefficients, or if the inconsistencies represent a limitation to be expanded on in the discussion.

THE UNIVERSITY OF CHICAGO | Biological Sciences Division

Seppe Kuehn Ph.D.
Associate Professor

Department of Ecology and Evolution
Center for the Physics of Evolving Systems
The University of Chicago
.
Gordon Center for Integrative Science
929 E. 57th St.
Chicago, IL 60637

607.351.2041
seppekuehn@uchicago.edu
kuehnlab.org

Please find enclosed a revision of our manuscript entitled *Epistatic interactions drive microbial community bioremediation and enable rational design* under consideration at Nature Microbiology (NMICROBIOL-25031102A-Z). Below we provide a step by step rebuttal and a higher level summary.

Summary: We performed the following substantial revisions:

1. A new amplicon sequencing experiment for ~12 communities before and after BPA degradation.
2. Significant edits to the text as recommended by the referees.
3. Modifications to Figure 1 for clarity.

A note on sequencing measurements: First, our strain library was selected on the basis of diversity in BPA degradation phenotypes and not taxonomic diversity. As a result, subsets of isolates are not reliably separable in the V3V4 hypervariable regions of the 16S gene (nor any other region of the gene). As a result, when we performed amplicon sequencing, we could not reliably disentangle abundances of these groups of taxa. This limitation of the sequencing measurement meant that for many strains (12 of 16) we could identify changes in relative abundances of individual taxa. As a result, we could not associate changes in relative abundances of individual strains (e.g. NL109) with their associated regression coefficients. Despite this limitation, the sequencing data did enable us to address several key questions from the referees (e.g. concerning degraders).

Second, our sequencing measurements revealed some contamination present in the strains used to assemble our synthetic communities. When we discovered this, we performed whole-genome long-read sequencing on all of our frozen stocks to quantify any and all contamination. As a result, some (4 of 16) of the previously reported “axenic” cultures used to initialize communities were not in fact axenic. Purifying these cultures and re-performing all of the BPA community experiments is beyond our human resource capabilities (Yousef is now completing the MD portion of an MD/PhD program). However, we believe this finding does not fundamentally change the findings of our study since the presence and absence of a strain in our communities simply reflects the presence and absence of these associated strains. Given the

reproducibility of our experiments at the level of BPA degradation dynamics, and the predictive power of our models, we believe the conclusions remain robust with this modification in interpretation. So we chose to carefully document and disclose this finding in the Methods and SI. We are *very grateful* that the reviewers requested the sequencing measurement because without this we would never have known and would have inadvertently reported incorrect methodologies! We would be much obliged to hear the thoughts of the referees regarding this aspect of the study and whether our addressing the issue in the paper is sufficient.

Sequencing data has been submitted to NCBI and is available in its entirety with all code and supporting data at the following Open Science Framework repository (https://osf.io/gcfr8/).

Below we present a detailed response to all of the referee requests.

Mahmoud Yousef & Seppe Kuehn

Reviewers Comments:

Reviewer #1 (Remarks to the Author):

In this manuscript, the authors designed bacterial communities for BPA degradation and investigated how degradation efficiency and functional landscapes can be deduced statistically from simple growth experiments at varying BPA concentrations. Working with novel isolates from the environment, they deduce that epistasis increases with increasing toxicity, meaning that collective effects become more important. However, while the overall approach is compelling, the findings hinge on the choice of analysis for the degradation (AUC) and could significantly benefit from some low-hanging fruits to assess the validity of the predictions. As I am no statistician, I do not comment on the appropriateness of the data or methods for the performed statistical analysis.

We thank the reviewer for their insightful comments which allowed us to improve the manuscript.

1. All statistical modeling is based on the area under the curve calculated from the degradation experiments. While numbers are shown in the Figures and AUC values can be downloaded, no information is given on how the AUC is calculated. Based on the figures and provided code, it seems that the authors did not normalize the AUC (e.g., as a fraction of the AUC where no degradation has occurred at all). If that is the case, it is not surprising that a higher initial BPA concentration generally results in a higher AUC since there is a dramatic baseline shift. In its current form, it is impossible to understand the sensitivity of the predictions to the choice of predictor variable.

While it is true that a higher BPA will trivially lead to a higher AUC, our statistical methods accounted for this through z-scoring (mean-centering and dividing by the standard deviation) every entry of each column (corresponding to a measurement within each BPA concentration). This makes the response variables for each concentration have a mean of 0 and a standard deviation of 1. This approach allows SoftImpute to be interpretable despite the differences in baseline, and enables a direct comparison of model coefficients despite different initial BPA concentrations. Our z-scoring approach was previously mentioned in the Methods, and we have expanded on this in the Results (in sections “Low-dimensional variation in community function” (lines 215-218) and “Learning the community-function landscape for BPA degradation” (lines 244-246).

We appreciate the consideration of the sensitivity of predictions to the predictor variable. This is a question we had considered. To address this we investigated two different metrics for BPA degradation – an estimate of the rate of BPA degradation and BPA concentration at a fixed time after inoculation. We found that these response variables yield the same qualitative results regarding epistasis and the role of strain NL109. This has been added to the Supplemental Information and the results are shown in Figure S8.

2. Some communities comprise far more species than others (but have an equal individual inoculum of OD 0.02), which changes the total inoculum size (e.g., mix8 must have fewer than 3 times as many bacteria compared to mix20 initially). Have the authors examined the impact of inoculum size on degradation rate/lag-phase?

Our regressions estimate the average impact of a strain on BPA degradation irrespective of the composition of the community. As a result of this, we wished to keep the initial abundance of every strain consistent across communities, such that community function is not affected by the *abundance* of the strains within it. For example, consider a hypothetical situation where each strain acts independently to degrade BPA but that degradation rate depends on the initial biomass. If we varied the initial biomass of each strain with community richness we would expect the contribution of each strain to decline with richness (due to starting at lower biomass). For this reason, we initialize each community with the same initial biomass of each strain. This allowed us to compare the impacts of each strain in a community-independent manner; as a result, we have not studied the impact of inoculum size on lag-phase. We have addressed this in the Methods, under the section “Synthetic Community Experiments” and we have added a brief comment to the results section (lines 152-156).

In addition, we would like to point out that community size is not the dominant factor in determining BPA degradation. In fact, Mix20 – our largest community – is not the fastest degrading community in any of the five initial BPA concentrations tested. We have added Figure S7 which examines the correlation between community size and

AUC. A significant correlation was found only at 60 ppm – where additive effects dominate BPA degradation – and 150 ppm – where a single strain exerts a strong additive effect. (see lines 336-343)

3. Understanding the individual contribution or growth of the species for each community is informative to benchmarking the model's performance. Assessing the absolute or relative abundance of the strains after the experiment and comparing them to the average effect of individual strains (beta) may provide a critical comparison to assess the model's effectiveness (if degradation translates to biomass increase). Currently, there is no way to gauge the performance of the model effectively.

We thank the reviewer for this comment. See comments above regarding our limited ability within the sequencing measurement to assess the dynamics of individual taxa. However, we did find another route to assessing the validity of some of the regression coefficient independently. In particular, we correlated the degradation of individual strains in monoculture with the additive regression coefficient for that strain in the community context. In the 60ppm condition, where the landscape is largely additive, we found a strong and significant correlation (new Figure S4). In contrast, at 150ppm we do not observe such a correlation due to limited degradation by any single strain in this condition. We feel that the observation in 60ppm of a strong correlation by individual strains in monoculture validates the regression coefficients (lines 292-295).

4. The authors included non-degraders in many of the communities, which I found intriguing. More information on their importance for degradation, and e.g., their final abundance, would be appreciated. I can see that the authors highlighted the non-degrader-non-degrader coefficients on page 16, but a deeper dive into their possible importance by looking at the final community composition (do they still show up?) would be helpful.

In response to this question we performed amplicon sequencing measurements on a few select communities prior to, and after, BPA degradation. Using a spike in method we measured absolute abundances, and our sequencing measurement permitted us to disambiguate degrader from non-degrader taxa. These data are summarized in the new Figure S5. What we observe is an increase in non-degrader absolute abundances in the 60ppm condition, supporting the idea that these strains participate in breakdown of intermediates. In some communities we observe a similar increase in the non-degrader population in 150ppm conditions as well. (see lines 319-328)

Minor comments:

1. In what way does the soft impute outperform simple interpolation for the different communities? If I understood correctly, soft impute predicts the degradation of an individual community at a specific concentration based on the observed degradation at other concentrations. How does that compare to simple regression or interpolation?

Figure S10 shows that SoftImpute performs on par, if not slightly worse, than regressions in predicting AUC. The relatively high performance for SoftImpute is not surprising given that SoftImpute is performing a simpler prediction than full regression (e.g. landscape inference), since it is given information about how each community performs at 4 other concentrations to predict performance at a target concentration. In contrast, regressions are only given the community composition to perform predictions. Thus comparing regression to softimpute is not a fair comparison since the latter is a more challenging statistical problem (see also lines 261-267).

Second, we are unclear as to what “simple interpolation” refers to. Figure S8 shows that predicting the mean for each concentration performs worse than both SoftImpute and regressions. Predicting the mean is perhaps the simplest version of an interpolation. We do note that the SoftImpute algorithm is essentially an interpolation in the sense that it is filling in missing data via a low-rank approximation to a matrix. This is analogous to interpolating a curve in one dimension, but instead for a matrix. We hope these comments clarify what we have done.

2. What is the reason for using log(AUC) instead of AUC as a predictor for the functional landscapes?

Log(AUC) is used to put all of the data on a similar scale across the five concentrations – making the range of our data between ~2.9 to ~4.1 instead of ~800 to ~11000. Log transformations are common in regression problems when the response variable changes over a very large range of values. When this happens the objective function (mean squared error) can be dominated by large values. The log-transformation avoids this. We have added a statement in the methods (lines 569-570), and under section “Learning the community-function landscape for BPA degradation” (lines 242-244), to address this.

3. Figure 2D: Despite similar degradation trends in the lower concentrations (e.g., 60 ppm and 80 ppm), there is a stark difference in the AUC. How would these look like with normalized AUC?

We implemented z-scoring during imputation, and our previous discussion of the approach addresses this. We mentioned this previously in the Methods, under section “Imputation to predict AUCs across concentrations of BPA”. We added a statement to section “Low-dimensional variation in community function” for further clarity (lines 215-218).

4. The concept of epistasis in terms of community interaction needs a clearer

introduction. Interpretation and translation to “community relevance” is difficult otherwise.

We appreciate the reviewer’s insight on the need to clarify the concept of epistasis. We had added a statement in the Introduction more cleanly introducing it in the context of community function (lines 61-64).

5. Figure 4D: This information may be valuable earlier in the manuscript.

We thank the author for this comment, and have moved this panel to Figure 1 (now Panel C).

6. Figure 5: Do the authors know why po109 (*now NO109*) seems to be important? Is it present in the final community? Also, since F1 is strongly increasing again from 125 ppm to 150 ppm, does the trend of increasing epistatic effects hold?

Please see our comment above regarding our inability to detect changes in NL109 via amplicon sequencing in communities. From our sequencing measurements we can say that the group of four strains that contain NL109 are present at the end of the experiment. We cannot conclusively say whether or not NL109 is included in that group.

As to the question of why NL109 is important. We do not have data that can definitively answer this question, although we suspect that NL109 is more resistant to BPA toxicity at higher concentrations.

The increasing F1 score can be attributed to the large additive coefficient from NL109 alone. You can see this clearly in the hatched portions of the bars in Figure S1. In this sense the increasing epistasis result holds when accounting for this single very large additive effect.

Reviewer #2 (Remarks to the Author):

This manuscript presents a well-structured study demonstrating the application of community-function landscape modeling to guide the rational design of synthetic microbial consortia. Using bisphenol A (BPA) degradation as a case study, the authors isolate a library of soil bacteria, quantify their degradation performance in multiple combinations and under varying contaminant concentrations, and construct statistical models that capture both additive and epistatic contributions to community function. They validate these predictions both in vitro and in soil microcosms, showing that the designed communities can outperform the native microbiota in degrading BPA. The study is conceptually innovative, methodologically sound, and offers a compelling proof of principle for predictive microbiome engineering.

The strengths of the work are the following. The experimental design is rigorous, with careful control over community composition and contaminant conditions. The modeling approach is clearly described and implemented, effectively capturing key features of microbial interaction networks that shape biodegradation outcomes. The ability to transition from in vitro predictions to performance validation in soil, a complex and more ecologically realistic environment, enhances the practical relevance of the findings. Overall, the manuscript is clearly written and presents a coherent narrative from hypothesis to validation.

We thank the reviewer for the positive assessment of our work and the careful reading leading to the helpful comments below.

Nonetheless, there are a limitations that merit deeper consideration in the discussion. First, while the soil experiments are a valuable step toward real-world application, natural environments are typically far more complex. Autochthonous microbial communities in diverse soils or aquatic systems may compete with, inhibit, or even displace introduced consortia. A more detailed discussion on how the inoculated synthetic community might be influenced by native microbiota and environmental variability would strengthen the translational perspective of the study.

We agree with the reviewer that natural environments are significantly more complex than the soil experiments we performed. The theory positied by *Silverstein et al* on niche specialization explains our success in this case, but we agree that our success cannot be generalized to the complicated natural microbiomes that exist. We have addressed this in the discussion as a limitation (lines 456-464).

Second, the exclusive focus on BPA as a single target contaminant limits the generalizability of the approach. In practice, polluted environments often contain mixtures of compounds—such as petroleum fractions may interact synergistically or antagonistically, complicating biodegradation. While it is reasonable to begin with a defined substrate, the authors should acknowledge that extending their method to multiple contaminants will pose additional challenges in both experimental design and modeling complexity.

We thank the reviewer for raising this point. Indeed, handling multiple pollutants simultaneously would introduce many computational and experimental difficulties, although we typically find environments polluted by many compounds simultaneously. Our approach would need to be extended to account for this, and we have added a discussion of this limitation in the discussion (lines 465-472).

Finally, the study does not report the composition of the synthetic consortia after the biodegradation experiments. Without data on the final community structure—whether through sequencing, plating, or another method—it is difficult to assess whether all

introduced strains remained active, or if certain taxa dominated the degradation process. If such analysis is not feasible at this stage, the authors should at least discuss this as a limitation.

We thank the reviewer for this comment, also raised by reviewer #1. The key advantage to our approach is that a community's initial composition is a sufficient predictor of community function. However, as discussed above, we have performed a sequencing experiment to quantify community composition at the beginning and end of the degradation experiment in minimal medium conditions. Despite the limitations of the sequencing measurements in these synthetic communities, we are able to show that non-degraders persist in the community. We believe that this measurement solidifies our initial results more fully. Please see responses to referee #1 regarding the details and conclusions of this sequencing experiment.
